# Supplementary material for: Reversible RNA phosphorylation stabilizes tRNA for cellular thermotolerance
Source: Nature. 2022 Apr 27;605(7909):372–9. doi: 10.1038/s41586-022-04677-2 (PMC9095486; doi:10.1038/s41586-022-04677-2)
Supplement: Supplementary file 1 — This file contains Supplementary Notes 1–7, Supplementary Figs. 1–10, Supplementary Tables 1–7 and the legend for Supplementary Video 1. [file 41586_2022_4677_MOESM1_ESM.pdf]

nature portfolio
https://doi.org/10.1038/s41586-022-04677-2

Supplementary information

Reversible RNA phosphorylation stabilizes tRNA for cellular thermotolerance

In the format provided by the authors and unedited

Supplementary information

Supplementary Notes

Supplementary Note 1 | Precise mapping of tRNA modification by mass spectrometry and detection of N^{324}

Total nucleoside analysis of the isolated $tRNA^{Val3}$ detected 13 species of RNA modifications (Extended Data Fig. 2a). We then precisely mapped all the modifications by analyzing RNA fragments digested by RNase T_1 (Extended Data Fig. 2b and Supplementary Table 1a) and RNase A (Extended Data Fig. 2c and Supplementary Table 1b). Ψ was converted to 1-cyanoethylpseudouridine ($ce^1\Psi$) to make it detectable by MS[54] (Supplementary Table 1c). In addition to $tRNA^{Val3}$ (Fig. 1a), we also detected $tRNA^{Val2}$ (Supplementary Fig. 1) , an isoacceptor with three substitutions in the anticodon loop. We then mapped 13 species of RNA modifications at 18 positions in both tRNAs (Fig. 1a and Supplementary Fig. 1). Only 1-methylguanosine (m^1G) was uniquely mapped at position 37 in $tRNA^{Val2}$ (Supplementary Fig. 1, Supplementary Table 1a,b). As archaeal- and/or thermophile-specific modifications, we detected ac^4C at position 6, G^+ at position 15, s^2U at position 54, and 1-methylinosine (m^1I) at position 57 in both tRNAs (Fig. 1a and Supplementary Fig. 1).

We detected an unknown uridine derivative with molecular mass 324, (tentatively named N^{324}) in the RNA fragments (Extended Data Fig. 2b,c). RNA-MS clearly detected unique RNA fragments bearing N^{324}: $N^{324}m^5Cm^5CUmGp$ (position 47–51, *m/z* 852.59, *z* = -2) (Extended Data Fig. 2b) and $GGAGGN^{324}m^5Cp$ (position 42–48, *m/z* 1,215.15, *z* = -2) (Extended Data Fig. 2c). The fragments were sequenced by CID, identifying N^{324} at position 47 (Fig. 1b,c). Because 2′-*O*-methyluridine at position 50 (Um50) (Fig. 1a) is a partial modification, we also detected four types of the RNA fragments with or without Um50 and N^{324}: $N^{324}m^5Cm^5CUmGp$, $N^{324}m^5Cm^5CUGp$, Um^5Cm^5CUmGp and Um^5Cm^5CUGp (Fig. 1d). The modification frequency of N^{324} was calculated to be 96.8% from the relative intensity of mass chromatograms (Fig. 1d).

To determine whether N^{324} was present in the other 11 tRNAs isolated from *S. tokodaii* (Extended Data Fig. 1), we digested each of the isolated tRNA with RNase T_1 and subjected the products to RNA-MS. We clearly detected N^{324} at position 47 of tRNAs for Lys2, Thr3, Gly3, Arg4, Pro2, Ile2, and Phe (Extended Data Fig. 3a and Supplementary

Table 2), but not in tRNAs for Gln2, Cys, Leu4, and Ser3 (Extended Data Fig. 3a and Supplementary Table 2). Each fragment was confirmed by CID (Extended Data Fig. 3b).

In addition to mature tRNAs, we successfully isolated precursor tRNAs for Ile2 and Phe bearing an intron (Extended Data Fig. 1). The N^{324}-containing fragments were present in both pre-tRNAs (Extended Data Fig. 3c), indicating that N^{324} is introduced in the precursor form. Compared to high frequency (82.1% and 94.3%) of N^{324} in mature tRNAs (Extended Data Fig. 3a), only 60.9% of pre-$tRNA^{Ile2}$ and 40.4% of pre-$tRNA^{Phe}$ are modified with N^{324} (Extended Data Fig. 3c). Because pre-tRNAs contain hypomodified nascent transcripts, it is considered that N^{324} is not efficiently introduced in such hypomodified precursors. In addition, extra sequences at both termini and intron of pre-tRNA might interfere N^{324} formation. Otherwise, hypomodified pre-tRNAs might be degraded rapidly by quality control system.

Supplementary Note 2 | Chemical structure determination of N^{324}

To our surprise, however, we did not detect N^{324} nucleoside in our LC/MS analysis (Extended Data Fig. 2a), whereas the other 13 species were clearly detected and mapped to the isolated tRNAs (Fig. 1a and Supplementary Fig. 1). High-resolution mass analysis of the N^{324}-containing fragment revealed that the additional mass of N^{324} attached to the uridine residue was 79.97067Da, equivalent to one phosphate group (theoretical mass 79.96632), with a low error value of 4.4 mmu, indicating that N^{324} is a phosphorylated uridine. This prediction nicely explains why the N^{324} nucleoside was not detected in our nucleoside analysis (Extended Data Fig. 2a), as N^{324} is dephosphorylated by the bacterial alkaline phosphatase (BAP) treatment to prepare total nucleosides. To confirm this observation, we treated the RNase T_1-digests of $tRNA^{Val3}$ with BAP, followed by RNA-MS analysis (Extended Data Fig. 4a). The RNA fragment bearing N^{324} ($N^{324}m^5Cm^5CUGp$) detected without BAP treatment was converted to the RNA fragment that lost two phosphates: one from 3′-phosphate and another from N^{324}. The fragment was assigned to $Um^5Cm^5CUG_{OH}$ based on the CID spectrum (Extended Data Fig. 4b).

To determine the phosphorylation site of N^{324}, we digested *S. tokodaii* tRNAs into total nucleotides using nuclease P_1 (Extended Data Fig. 4c) and subjected the products to LC/MS/MS analysis. We detected a dinucleotide $pN^{324}m^5C$ (m/z 722, z = -1) (Extended Data Fig. 4d), indicating that N^{324} hinders 3′-cleavage by nuclease P_1. The dinucleotide was further probed by CID (Extended Data Fig. 4e). By assigning the product ions, we detected a product ion missing

the uracil base (*m/z* 610) (Extended Data Fig. 4e), strongly indicating that the uracil base of N^{324} remains unmodified. Thus, the phosphate group should be attached to the ribose moiety. Furthermore, the dinucleotide was subjected to periodate oxidation followed by β-elimination to remove m^5C48, yielding the 5′, 3′-diphosphonucleotide ($pN^{324}p$, *m/z* 483, $z = -1$) (Extended Data Fig. 4f). The CID of this nucleotide confirmed the loss of the uracil base (*m/z* 371) (Extended Data Fig. 4f). If phosphorylation occurs in the ribose moiety, the 2′-OH group is the most plausible position (Extended Data Fig. 4f). To confirm this hypothesis, we treated $tRNA^{Val3}$ with yeast Tpt1p, a 2′-phosphotransferase that removes 2′-phosphate groups from RNA [33,69]. The reaction product was digested with RNase T_1 and subjected to RNA-MS (Extended Data Fig. 4g). The RNA fragment with N^{324} ($N^{324}m^5Cm^5CUGp$) was completely dephosphorylated and converted to a fragment with U (Um^5Cm^5CUGp) (Extended Data Fig. 4g). The dephosphorylated fragment was confirmed by CID (Extended Data Fig. 4h). Taken together, these observations confirm that N^{324} is 2′-phosphouridine (denoted U^p, "p" is a superscript to discriminate it from 3′-phosphate) (Fig. 1e).

Supplementary Note 3 | Structural characterization of tRNA modifications

We report here for the first time the atomic structure of a native archaeal tRNA with thermophile-specific post-transcriptional modifications including U^p47, ac^4C6 and G^+15, revealing the molecular basis of structural stabilization mediated by tRNA modifications. Among 18 tRNA modifications, we observed clear electron densities for 15, including U^p47 (Fig. 2a and Extended Data Fig. 5c,d), G^+15 (Extended Data Fig. 5e,f), ac^4C6 (Extended Data Fig. 5g,h), m^1I57 (Extended Data Fig. 5i,j), m^1A58 (Extended Data Fig. 5i,j), and $m^{2,2}G26$ (Extended Data Fig. 5k,l), whereas s^4U8, Um50, and s^2U54 were not visible due to their low modification frequencies.

G^+15 is a unique modification found in archaeal tRNAs that contributes to the thermal stability of tRNA [7,19,23]. G^+15 forms a Levitt base pair with m^5C48 on the top of base-triple layers in the tRNA core (Fig. 2b,c, Extended Data Fig. 5e,f), and the two nitrogen atoms of the amidine group interact with anionic phosphate oxygen atoms of the backbones of G7, A14, and G^+15 (Extended Data Fig. 5e). In yeast $tRNA^{Phe}$, the hydrated manganese ion replaces the role of G^+15 (Supplementary Fig. 2a,b) [29]. G^+15 stabilizes the tRNA core structure by connecting the D- and V-loops to the acceptor stem without help of a divalent cation.

ac^{4}C at position 6 is also clearly visible (Extended Data Fig. 5g). This modification favors a C3′-*endo* form [17], contributing to the structural rigidity of RNA and decoding fidelity [16,70,71]. ac^{4}C6–G67 pair is expected to strengthen the acceptor stem (Extended Data Fig. 5g,h). The acetyl group of ac^{4}C6 protrudes into the major groove and orients proximal to C5 (Extended Data Fig. 5h), enabling ac^{4}C6 to stack with the neighboring base pair.

m^{1}I at position 57 (Extended Data Fig. 5i) is another unique modification found in archaeal tRNAs [10,72,73]. The D- and T-loop interaction is stabilized by several modifications (Extended Data Fig. 5j). m^{1}I57 is sandwiched between the G18–Ψ55 and G19–Cm56 pairs. The N7 of m^{1}I57 forms a hydrogen bond with 2′-OH of Ψ55 (Extended Data Fig. 5j), and the m^{1}I57 base contributes to base stacking with the neighboring base pairs. s^{2}U54 pairs with m^{1}A58 by reverse-Hoogsteen base-pairing [9,74]. s^{2}U54 favors the C3′-*endo* form due to steric repulsion between the 2-thio and 2′-OH groups [11], stabilizing the s^{2}U54–m^{1}A58 pair (Extended Data Fig. 5j).

Supplementary Note 4 | U^{p}47 is widely distributed in archaea

We examined the phylogenetic distribution of the U^{p}47 modification in archaea. For this purpose, we prepared tRNA fractions from eight archaeal species including four crenarchaea: *S. acidocaldarius*, *S. solfataricus*, *Aeropyrum pernix*, and *Pyrobaculum oguniense*; three euryarchaea: *T. kodakarensis*, *Methanosarcina acetivorans*, and *Thermoplasma acidophilum*; and one thaumarchaeon: *Nitrososphaera viennensis*. The tRNA fraction from each species was digested by nuclease P$_1$ and subjected to LC/MS analysis to detect pU^{p}m^{5}C (m/z 724, z = 1) (Extended Data Fig. 8a). In this analysis, U^{p}47 was detected in tRNAs from *S. acidocaldarius*, *S. solfataricus*, *A. pernix*, and *T. kodakarensis*, but not in those from *M. acetivorans* (Extended Data Fig. 8a). For *N. viennensis*, *P. oguniense*, and *T. acidophilum*, we could not obtain sufficient tRNA fraction for nucleotide analysis. Instead, the sample was digested by RNase T$_1$, and the RNA fragments were subjected to highly sensitive analysis using capillary-LC/nanoESI-MS (shotgun analysis) to detect RNA fragments containing U47 with or without 2′-phosphate. We clearly detected the U^{p}47-containing fragments in the tRNA fractions from *N. viennensis* (Extended Data Fig. 8b) and *P. oguniense* (Extended Data Fig. 8c), which were sequenced by CID (Extended Data Fig. 8b,c). We also confirmed disappearance of the U^{p}47-containing fragments following Tpt1p treatment (Extended Data Fig. 8b,c). In *T. acidophilum*, we detected U47-containing fragments without 2′-phosphorylation (Extended Data Fig. 8d).

Taken together, these observations demonstrate that U^{p}47 is present in six species, but absent in *M. acetivorans* and *T. acidophilum*.

Supplementary Note 5 | Characterization of ArkI homologs

According to the phylogenetic distribution (Supplementary Fig. 4), ArkI homologs are mainly found in thermophilic archaeal species but are also present in some thermophilic bacteria, including *Aquifex aeolicus* and *Nautilia profundicola,* as well as in mesophilic archaea and bacteria. We also obtained recombinant proteins of the other ArkI homologs and examined *in vitro* U^{p}47 formation. We confirmed the activity of ArkI homologs from two archaeal species, *Methanocaldococcus fervens*, and *N. viennensis* (Supplementary Fig. 5a), as well as two bacterial species, *A. aeolicus* and *N. profundicola* (Supplementary Fig. 5a).

We then carried out *in vitro* phosphorylation of total RNA by ArkI homologs (Supplementary Fig. 5b,c). Only class I tRNA fraction was phosphorylated, whereas no other RNAs including class II tRNAs and rRNAs were phosphorylated, indicating that ArkI homologs have strict substrate specificity.

Supplementary Note 6 | Characterization of ligands that bind to TkArkI

To analyze the ligand, we extracted small molecules from recombinant TkArkI and analyzed them by LC/MS (Supplementary Fig. 7a,b). Guanosine was clearly detected as the most abundant ligand, and deoxyguanosine as the second major ligand. Although a trace amount of adenosine was detected (Supplementary Fig. 7a), unexpectedly, neither ATP nor ADP was observed. This finding prompted us to examine whether GTP can be used for 2′-phosphorylation (Supplementary Fig. 7c). Although weak activity was seen in the presence of GTP, we concluded that ATP is an appropriate phosphate donor for U^{p}47 formation.

Supplementary Note 7 | Phosphorylation of proteins and RNAs

Phosphorylation, an enzymatic reaction that frequently occurs in proteins and metabolites, is involved in various biological processes, including signal transduction, metabolism, anabolism, and respiration via dynamic regulation mediated by kinases and phosphatases [47,48,75,76]. RNA molecules are also phosphorylated, but in most cases at their 5′ and 3′ termini. Polynucleotide kinase of T4 phage phosphorylates the 5′ end of the tRNA half fragment to repair the tRNA [77]. hClp1 is a kinase that phosphorylates the 5′ end of RNA and contributes to

pre-tRNA processing [78], phosphorylation of siRNA [79] and miRNA [80], and mRNA 3′ end formation [81]. Grc3 [82] and Nol9 [83] are polynucleotide 5′-kinases involved in rRNA processing. Recent work showed that RelA-SpoT homolog toxins pyrophosphorylate the 3′ ends of tRNAs to inhibit protein synthesis [84]. As an example of phosphorylation of internal RNA residue, the tRNA-modifying enzyme TiaS activates the cytosine base at the target site by phosphorylation to form a transient intermediate, thereby synthesizing agmatidine (agm2C) in tRNA [85,86].

Supplementary Figures

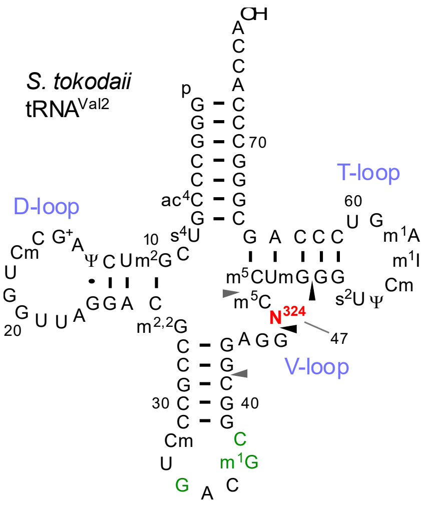

Supplementary Fig. 1 | Secondary structure of *S. tokodaii* tRNA^{Val2} with post-transcriptional modifications.

N^{324} is shown in red. Residues that differ between tRNA^{Val2} and tRNA^{Val3} are shown in green. The cleavage sites of RNase T_1 and RNase A that generate the RNA fragment containing N^{324} are indicated by black and gray triangles, respectively.

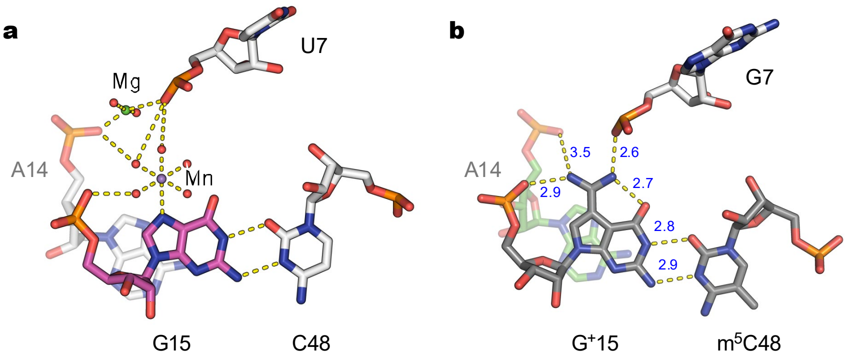

Supplementary Fig. 2 | Comparison of atomic structures of G^{+}15 and unmodified base.
(a) Levitt base pair of G15 and C48 in *S. cerevisiae* tRNA^{Phe} (1EHZ). G15 coordinates the hydrated manganese ion (Mn) and magnesium ion (Mg) to stabilize the core region by hydrogen bonding with the neighboring residues.
(b) Levitt base pair of G^{+}15 and m^{5}C48 with neighboring residues in *S. tokodaii* tRNA^{Val3}. Dashed lines indicate predicted interactions with bond length in Å.

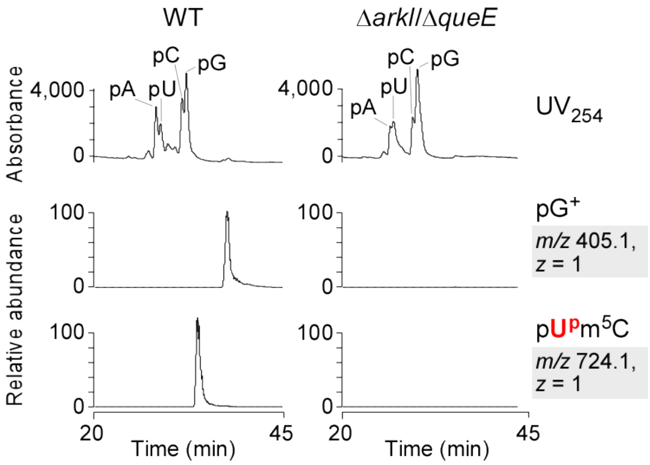

Supplementary Fig. 3 | Absence of G^+ and U^p in Δ*arkI*/Δ*queE* strain.
LC/MS nucleotide analysis of tRNA fractions from WT (left panels) and Δ*arkI*/Δ*queE* (right panels) strains of *T. kodakarensis*. Top panels show UV trace at 254 nm. The peaks for pA, pU, pC, and pG are marked. XICs show the proton adducts of pG^+ (*m/z* 405.1, *z* = 1, middle panels) and pU^pm^5C (*m/z* 724.1, *z* = 1, bottom panels) are shown.

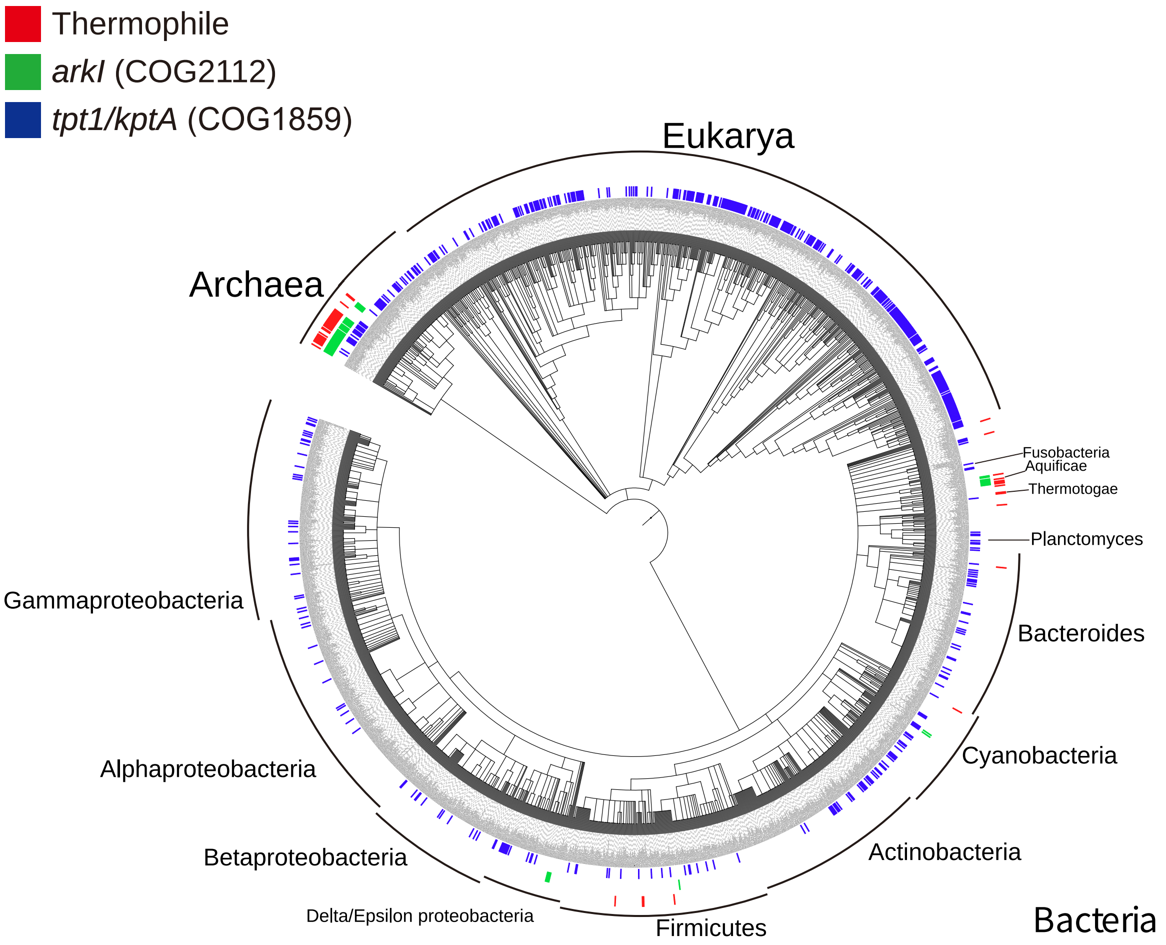

Supplementary Fig. 4 | Phylogenetic distribution of *arkI* and *tpt1/kptA*.
Orthologs of *arkI* (green rims) and *tpt1/kptA* (blue rims) in all domains of life were obtained from the IMG database (https://img.jgi.doe.gov/cgibin/w/main.cgi). The phylogenic tree was made using PhyloT (http://phylot.biobyte.de/) and visualized using iTOL (http://itol.embl.de). Thermophiles (red rims) were obtained from Bacdive (https://bacdive.dsmz.de/) and Genome Online Database (https://gold.jgi.doe.gov/).

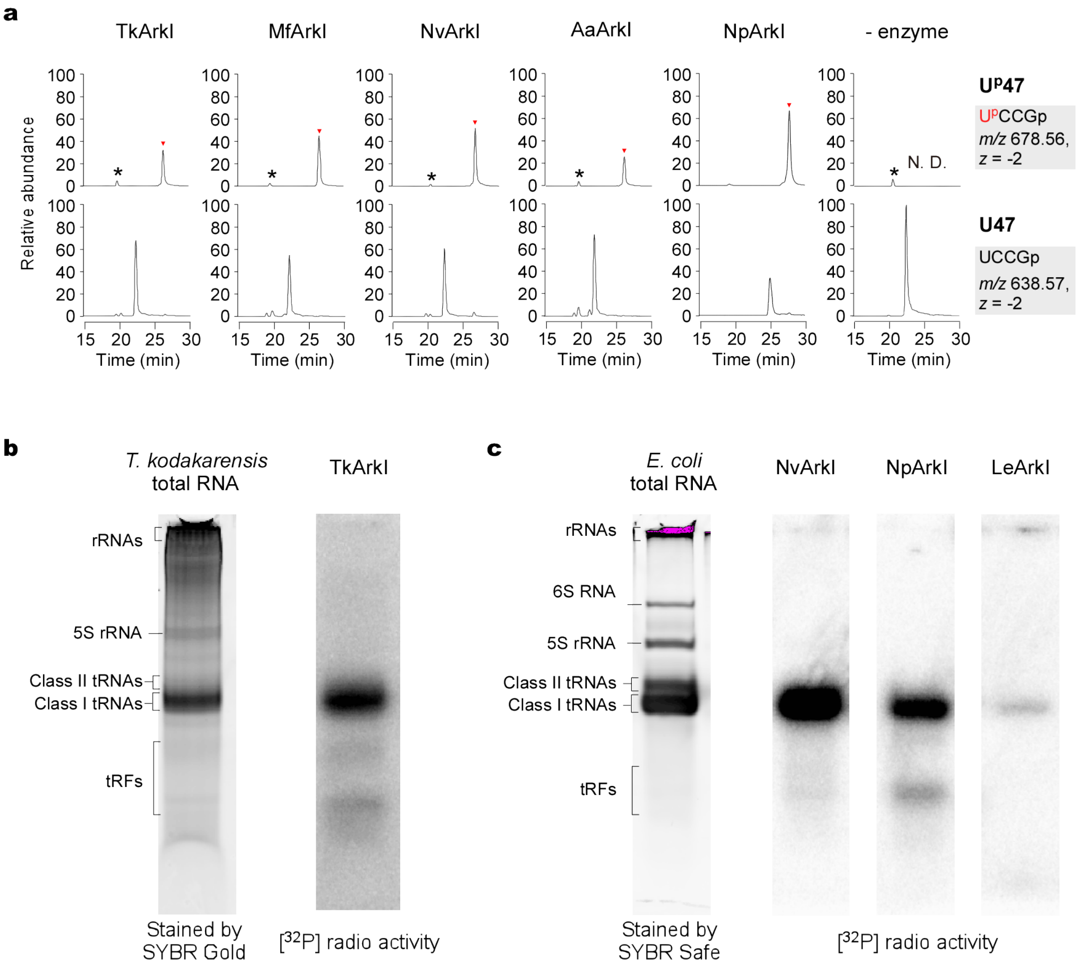

Supplementary Fig. 5 | *In vitro* U^{p}47 formation by ArkI homologs.
(a) *In vitro* U^{p}47 formation was examined by recombinant proteins of ArkI homologs from *T. kodakarensis*, *M. fervens*, *N. viennensis*, *A. aeolicus*, and *N. profundicola*. XICs show divalent negative ions of the RNase T_1-digested fragment containing U^{p}47 (upper panels) or U47 (lower panels). U^{p}47-containing fragments are indicated by red triangles. Unassigned fragments are indicated by asterisks.
(b) *In vitro* phosphorylation of *T. kodakarensis* total RNA (from Δ*arkI* strain) by TkArkI. After the reaction, total RNA was resolved by PAGE (left panel), and ^{32}P-labeled RNAs were visualized by fluoroimager (right panel). Total RNA stained by SYBR Safe was loaded as a marker. 5S rRNA (126 nt), Class II tRNAs (86-88 nt), Class I tRNAs (75-78 nt) are indicated. We confirmed the reproducibility of this result. The unprocessed gel image is provided in Supplementary Data 10.
(c) *In vitro* phosphorylation of *E. coli* total RNA by ArkI homologs (NvArkI, NpArkI and LeArkI). After the reaction, total RNA was resolved by PAGE (leftmost panel), and ^{32}P-labeled RNAs were visualized by fluoroimager (right panels). Total RNA was loaded as a marker. 6S RNA (183 nt), 5S rRNA (120 nt), Class II tRNAs (85-95 nt), Class I tRNAs (74-77 nt) are indicated. We confirmed the reproducibility of this result. The unprocessed gel image is provided in Supplementary Data 10.

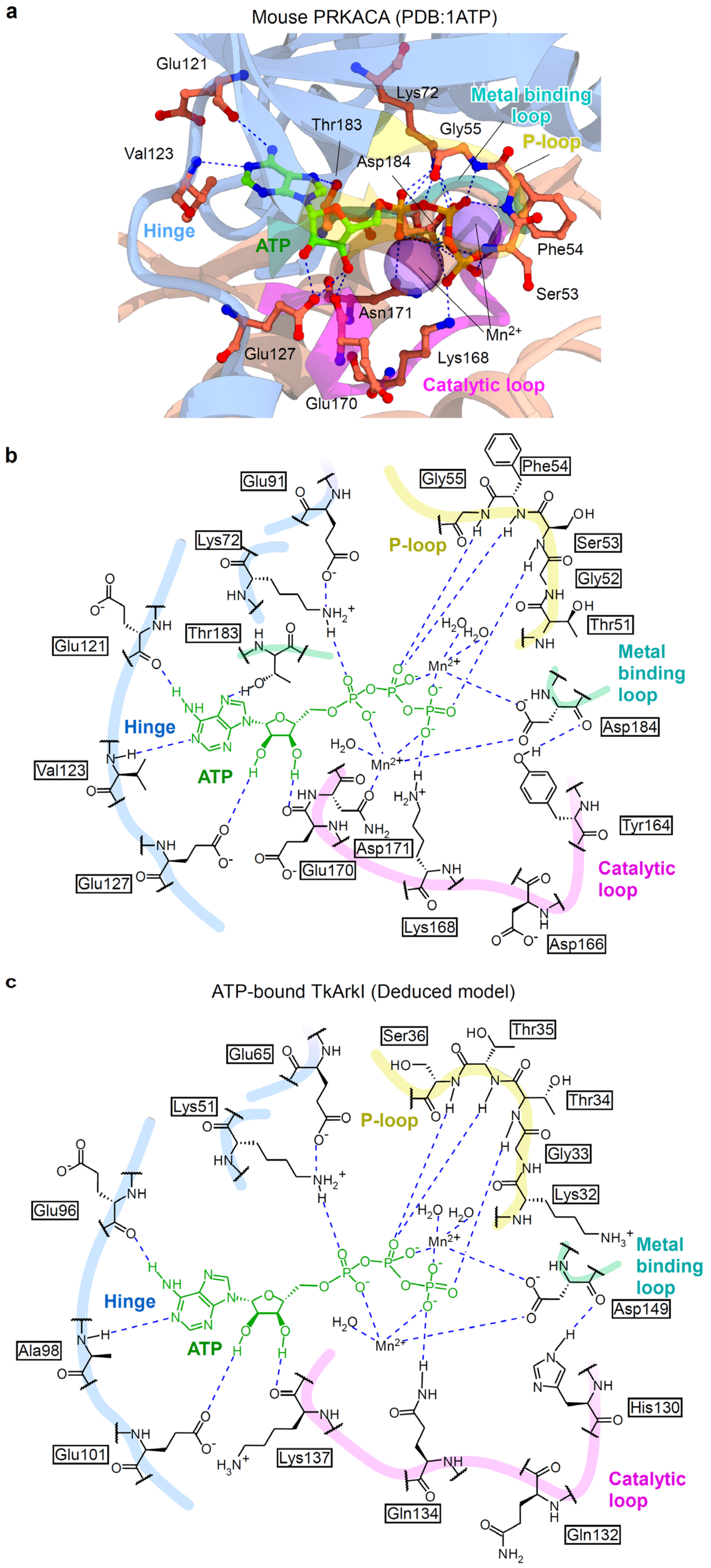
a
Mouse PRKACA (PDB:1ATP)
Glu121
Val123
Thr183
Lys72
Metal binding loop
Gly55
P-loop
Asp184
Hinge
ATP
Phe54
Ser53
Asn171
Mn2+
Glu127
Lys168
Catalytic loop
Glu170
b
Glu91
Gly55
Phe54
Lys72
P-loop
Ser53
Gly52
Glu121
Thr183
Thr51
Metal binding loop
Hinge
Asp184
Val123
ATP
Tyr164
Asp171
Glu170
Catalytic loop
Glu127
Lys168
Asp166
c
ATP-bound TkArkI (Deduced model)
Ser36
Thr35
Glu65
Lys51
P-loop
Thr34
Gly33
Glu96
Lys32
Metal binding loop
Hinge
Asp149
Ala98
ATP
His130
Glu101
Lys137
Catalytic loop
Gln134
Gln132

Supplementary Fig. 6 | ATP-bound model of TkArkI active site deduced from ePK.
(a) Close-up view of the ATP-binding site of mouse cAMP-dependent protein kinase catalytic subunit alpha (PRKACA, PDB: 1ATP). ATP and amino acid residues involved in ATP binding are shown in ball-and-stick representation. Manganese ions are shown as spheres. The color code for each motif is the same as in Fig. 4c.
(b) A schematic diagram of the ATP-binding site of mouse PRKACA. Predicted interactions are indicated by dashed lines. The main chains of P-loop, hinge, catalytic loop, and metal-binding loop are shown as bold lines with the same color codes as in Fig. 4d.
(c) A schematic diagram of putative ATP-binding model of TkArkI deduced from the PRKACA active site. Predicted interactions are indicated by dashed lines. The main chains of P-loop, hinge, catalytic loop, and metal-binding loop are shown with bold lines with the same color codes as in Fig. 4d.

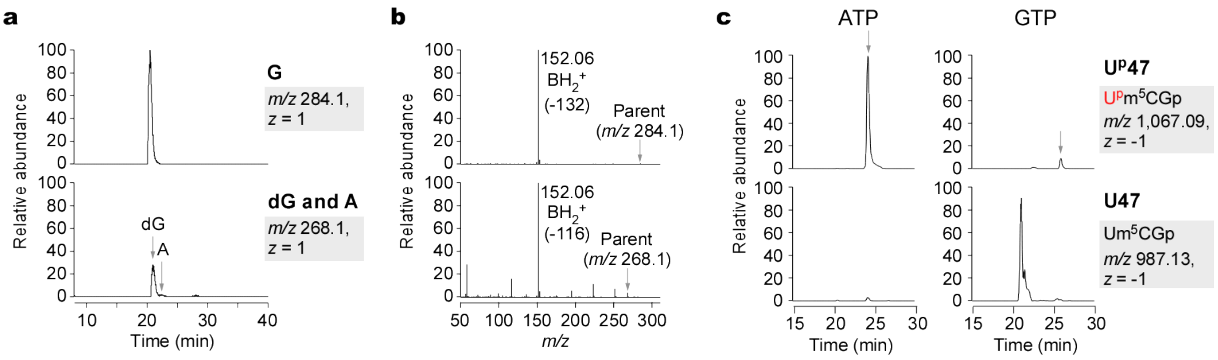

Supplementary Fig. 7 | LC/MS analysis of the ligands bound to TkArkI.
(a) XICs detecting proton adducts of guanosine, G (*m/z* 284.1, *z* = 1, upper panel) and deoxyguanosine, dG or adenosine, A (*m/z* 268.1, *z* = 1, lower panel). dG and A are indicated by grey arrows.
(b) CID spectra for guanosine (upper panel) and deoxyguanosine (lower panel). The same base-related ions (BH^{2+}) were detected from both spectra. Each parent ion is indicated by grey arrows.
(c) *In vitro* U^p47 formation by TkArkI in the presence of ATP or GTP. U^p-containing fragments are indicated by grey arrows.

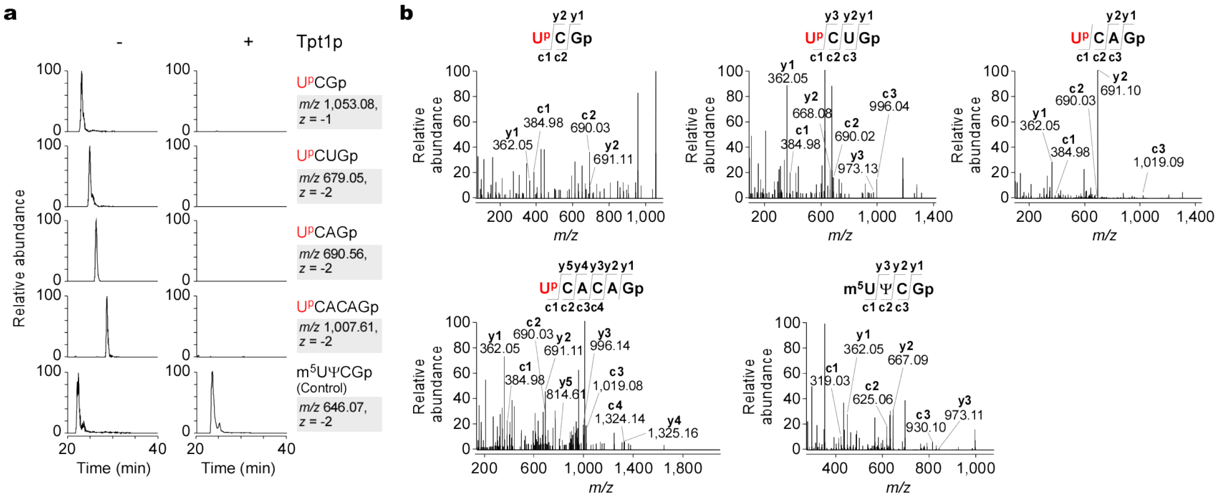

Supplementary Fig. 8 | Confirmation of U^p^47 introduced to *E. coli* tRNAs.

(a) Tpt1p-treatment of four U^p^47-containing fragments in tRNA fraction from *E. coli* Δ*trmB*/Δ*tapT* strain expressing *N. viennensis* ArkI. XICs show monovalent or divalent negative ions of U^p^CGp (top panels), U^p^CUGp (second panels), U^p^CAGp (third panels), U^p^CACAGp (fourth panels), and m^5UΨCGp as a control fragment (bottom panels) before (-) (left panels) and after (+) (right panels) Tpt1p-treatment.

(b) CID spectra of four U^p^47-containing fragments and a control fragment from *E. coli* tRNAs. Product ions are assigned in each fragment.

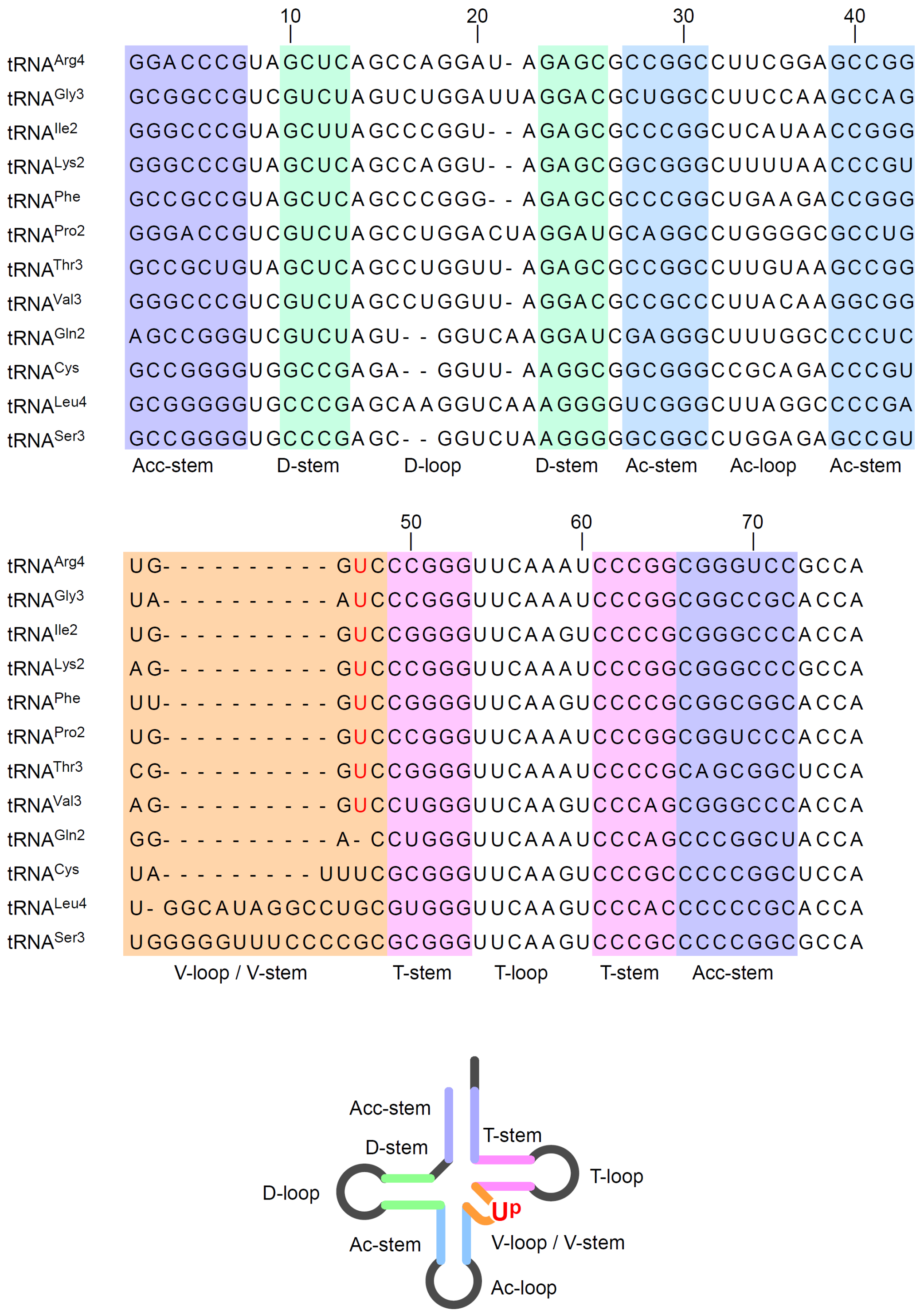

Supplementary Fig. 9 | Primary sequences of *S. tokodaii* tRNAs analyzed in this study. Sequences in the stem regions and V-loop are colored as indicated in the cartoon. U47 to be modified by ArkI is colored in red.

Fig. 1g

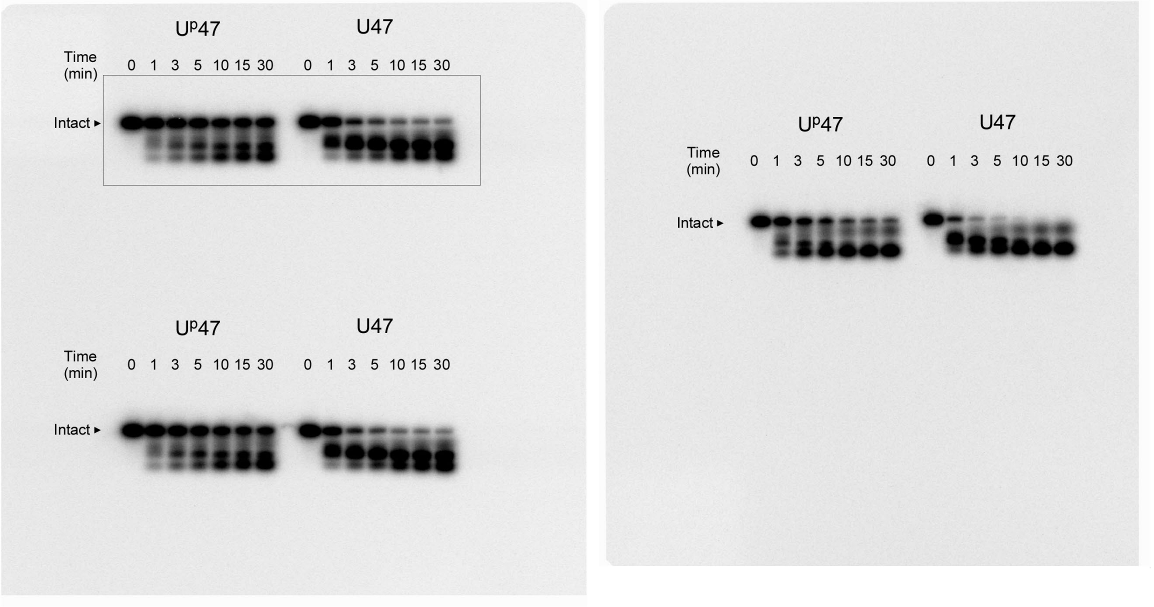

Extended Data Fig. 1

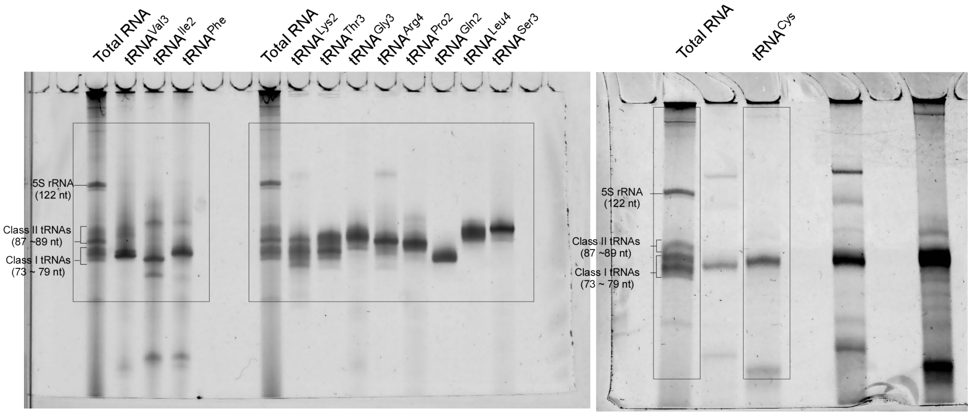

Supplementary Fig. 5b

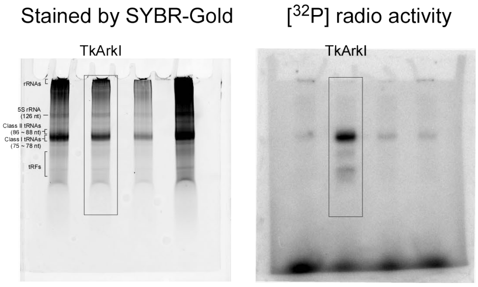

Supplementary Fig. 5c

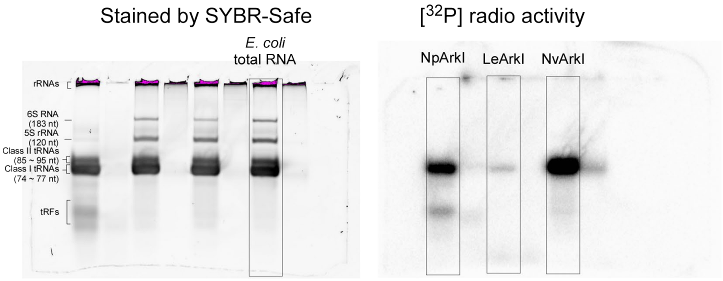

Supplementary Fig. 10 | Unprocessed gel images.

Supplementary Tables

Supplementary Table 1a | RNase T$_1$-digested RNA fragments of *S. tokodaii* tRNA$^{Val2/3}$.

No.	Sequence of RNA fragment	Position	Molecular Mass	Monoisotopic *m/z*		Charge state
				Calculated	Observed	
1	CCCmUUmACAAGp	30 : 39	3,210.463	1,069.146	1,069.148	-3
2	UCΨAG^{+}CCmUGp	11 : 18	2,925.400	974.125	974.128	-3
3	CCCACCA$_{OH}$	70 : 76	2,121.356	1,059.670	1,059.674	-2
4a	s^{2}UΨCmm^{1}Im^{1}AGp	54 : 59	1,997.263	997.624	997.625	-2
4b	UΨCmm^{1}Im^{1}AGp	54 : 59	1,981.286	989.635	989.639	-2
5	UCCCAGp	60 : 65	1,913.260	955.622	955.625	-2
6a	N^{324}m^{5}Cm^{5}CUmGp	47 : 51	1,707.204	852.594	852.596	-2
6b	N^{324}m^{5}Cm^{5}CUGp	47 : 51	1,693.189	845.587	845.589	-2
6c	Um^{5}Cm^{5}CUmGp	47 : 51	1,627.238	812.611	812.613	-2
6d	Um^{5}Cm^{5}CUGp	47 : 51	1,613.222	805.603	805.605	-2
7	CCCmUGp (tRNA^{Val2})	30 : 34	1,598.223	798.104	798.106	-2
8	CCac^{4}CGp	4 : 7	1,320.192	1,319.185	1,319.186	-1
9	UUAGp	20 : 22	1,304.161	1,303.153	1,303.157	-1
10a	ACm^{2,2}G>p	24 : 26	1,007.170	1,006.165	1,006.168	-1
10b	ACm^{2}G>p or<br>ACm^{1}G>p (tRNA^{Val2})	24 : 26<br>35 : 37	993.157	992.149	992.150	-1
11a	s^{4}UCm^{2}Gp	8 : 10	1,004.117	1,003.110	1,003.112	-1
11b	UCm^{2}Gp	8 : 10	988.140	987.132	987.134	-1
12	CCGp	27 :29	973.140	972.133	972.137	-1
13	AGp	44 : 45	692.111	691.103	691.104	-1
14	CGp	41 : 42<br>66 : 67	668.099	667.091	667.093	-1

Supplementary Table 1b | RNase A-digested RNA fragments of *S. tokodaii* tRNA$^{Val2/3}$.

No.	Sequence of RNA fragment	Position	Molecular Mass	Monoisotopic *m/z*		Charge state
				Calculated	Observed	
1a	GGAGGN^{324}m^{5}Cp	42 : 48	2,432.301	1,215.143	1,215.148	-2
1b	GGAGGUp	42 : 47	2,033.278	1,015.631	1,015.614	-2
2a	UmGGGs^{2}UΨp	50 : 55	2,001.222	999.603	999.607	-2
2b	UmGGGs^{2}Up	50 : 54	1,695.196	846.590	846.593	-2
2c	UmGGGUp	50 : 54	1,679.219	838.602	838.604	-2
2d	GGGs^{2}UΨp	51 : 55	1,681.181	839.582	839.588	-2
2e	GGGUp	51 : 54	1,359.178	678.581	678.586	-2
3	Cmm^{1}Im^{1}AGUp	56 : 60	1,675.261	836.622	836.626	-2
4	AAGGCp	37 : 41	1,671.252	834.618	834.623	-2
5	AGGACp	21 : 25	1,671.252	834.618	834.624	-2
6	pGGGCp	1 : 4	1,438.160	718.072	718.075	-2
7	GGGCp	67 :70	1,358.194	678.089	678.091	-2
8	AG^{+}Cp	14 :16	1,038.178	1,037.171	1,037.174	-1
9	GGUp	18 : 20	1,014.131	1,013.123	1,013.125	-1
10	GGCp (tRNA^{Val2})	39 : 41	1,013.147	1,012.139	1,012.143	-1
11	AGCp	64 : 66	997.152	996.144	996.149	-1
12	GACp (tRNA^{Val2})	34 : 36	997.152	996.144	996.145	-1
13	UmACp	34 : 36	972.145	971.137	971.141	-1
14	m^{2,2}GCp	26 : 27	696.131	695.123	695.122	-1
15a	Gs^{4}Up	7 : 8	685.060	684.053	684.051	-1
15b	GUp	7 : 8	669.083	668.075	668.077	-1
16	m^{2}GUp	10 : 11	683.099	682.091	682.092	-1
17	m^{1}GCp (tRNA^{Val2})	37 : 38	682.115	681.107	681.107	-1
18	GCp	29 : 30	668.099	667.091	667.093	-1
19	ACp	73 : 74	652.104	651.097	651.098	-1
20	CmUp	17 : 17a<br>32 : 33	643.093	642.085	642.089	-1

Supplementary Table 1c | Cyanoethylated RNA fragments of *S. tokodaii* tRNA$^{Val2/3}$.

No.	Sequence of RNA fragment	Position	Molecular Mass	Monoisotopic *m/z*		Charge state
				Calculated	Observed	
1	UC(Ψ+CE)AG^{+}CCmUGp	11 : 18	2,978.427	991.801	991.812	-3
2	s^{2}U(Ψ+CE)Cmm^{1}Im^{1}AGp	54 : 59	2,050.290	1,024.137	1,024.142	-2

Supplementary Table 2 | RNase T_1-digested fragments containing V-loop regions of *S. tokodaii* tRNAs.

tRNA	Type	Position 47	Length of V-loop (nt)	Sequence of RNA fragment	Position	Molecular Mass	Monoisotopic *m/z*	Charge state
tRNAs possessing U^{p}47								
tRNA^{Gly3}	class I	U^{p}	5	UAAU^{p}m^{5}Cm^{5}CCmGp	44 : 51	2,670.351	889.109	-3
		U		UAAUm^{5}Cm^{5}CCmGp	44 : 51	2,590.384	862.454	-3
tRNA^{Pro2}, tRNA^{Arg4}, tRNA^{Lys2}	class I	U^{p}	5	U^{p}m^{5}Cm^{5}CCmGp,	47 : 51	1,706.220	852.100	-2
		U		Um^{5}Cm^{5}CCmGp	47 : 51	1,626.254	812.120	-2
tRNA^{Thr3}, tRNA^{Phe}, tRNA^{Ile2}	class I	U^{p}	5	U^{p}m^{5}Cm^{5}CGp	47 : 50	1,386.156	692.570	-2
		U		Um^{5}Cm^{5}CGp	47 : 50	1,307.197	652.590	-2
tRNAs lacking U^{p}47								
tRNA^{Cys}	class I	U	6	UUAUUUm^{5}CGp	43 : 49	2,541.294	1,269.639	-2
tRNA^{Gln2}	class I	-	4	Am^{5}Cm^{5}CUGp	46 : 51	1,636.250	817.117	-2
tRNA^{Leu4}	class II	methylated G	long	CCU(G+2me)(C+me)Gp	e22 : 49	1,971.302	984.643	-2
tRNA^{Ser3}	class II	methylated G	long	UUUCCCC(GC+2me)Gp	e2 : 49	3,179.419	1,058.799	-3

Supplementary Table 3a | Torsion angles and ribose puckering in *S. tokodaii* tRNA^{Val3} Molecule A.

Position	Nucleotide	α	β	γ	δ	ε	ζ	χ	puckering
1	G	-	150	48	80	-143	-83	-178	C3′-*endo*
2	G	-54	170	48	78	-154	-66	-169	C3′-*endo*
3	G	-70	-178	56	78	-146	-74	-163	C3′-*endo*
4	C	-55	164	57	78	-147	-66	-163	C3′-*endo*
5	C	-68	164	71	72	-152	-80	-163	C3′-*endo*
6	ac^{4}C	-56	156	68	83	-150	-72	-165	C3′-*endo*
7	G	-63	-179	59	140	-89	-55	-135	C2′-*endo*
8	s^{4}U	-73	-163	58	84	-116	-134	-160	C3′-*endo*
9	C	128	-171	-143	127	-100	-61	-122	C1′-*exo*
10	m^{2}G	164	-162	70	84	-144	-80	-178	C3′-*endo*
11	U	-54	174	43	84	-150	-72	-160	C3′-*endo*
12	C	-60	172	52	79	-147	-72	-152	C3′-*endo*
13	Ψ	121	-142	-151	87	-154	-95	-172	C3′-*endo*
14	A	100	-177	-123	91	-125	-61	-172	C2′-*exo*
15	G$^+$	-45	156	53	87	-119	-76	-155	C3′-*endo*
16	C	-50	169	51	143	-93	-71	-112	C2′-*endo*
17	Cm	-52	156	54	78	-155	56	-148	C3′-*endo*
17a	U	166	152	39	148	-85	-122	-131	C2′-*endo*
18	G	59	162	44	151	-139	-101	-115	C2′-*endo*
19	G	-61	-166	34	145	-87	-143	-76	C2′-*endo*
20	U	-179	163	170	151	-90	-63	-129	C2′-*endo*
20a	U	-67	169	-52	155	-119	-100	-109	C2′-*endo*
21	A	75	175	66	145	-130	-82	-130	C2′-*endo*
22	G	-173	86	127	79	-132	-87	-179	C3′-*endo*
23	G	-42	164	41	77	-148	-78	-164	C3′-*endo*
24	A	-56	167	51	82	-142	-70	-161	C3′-*endo*
25	C	-63	165	51	78	-147	-67	-165	C3′-*endo*
26	m^{2,2}G	-58	168	53	79	-146	-67	-172	C3′-*endo*
27	C	-67	-174	51	80	-140	-79	-170	C3′-*endo*
28	C	-63	171	49	79	-144	-78	-165	C3′-*endo*
29	G	-58	165	55	78	-150	-77	-169	C3′-*endo*
30	C	-56	168	52	79	-152	-70	-166	C3′-*endo*
31	C	-65	178	54	82	-142	-65	-162	C3′-*endo*
32	Cm	-80	169	64	80	-126	-66	-156	C3′-*endo*
33	U	-79	-178	55	80	-130	-86	-148	C3′-*endo*

34	Um	150	-173	62	84	-135	-70	-167	C3′-*endo*
35	A	-56	169	45	81	-158	-101	-145	C3′-*endo*
36	C	143	-134	170	79	-121	-75	-164	C3′-*endo*
37	A	-52	174	43	81	-138	-76	-178	C3′-*endo*
38	A	-55	160	56	83	-134	-60	-179	C3′-*endo*
39	G	-65	177	47	80	-152	-75	-168	C3′-*endo*
40	G	-65	173	56	82	-150	-71	-165	C3′-*endo*
41	C	-62	172	53	77	-153	-72	-163	C3′-*endo*
42	G	-62	176	50	75	-149	-75	-165	C3′-*endo*
43	G	-49	165	52	78	-146	-87	-163	C3′-*endo*
44	A	-45	155	54	82	-141	-65	-163	C3′-*endo*
45	G	-63	172	47	83	-134	-95	-157	C3′-*endo*
46	G	-62	-165	59	84	-130	-70	-119	C3′-*endo*
47	U^p	-51	166	69	139	-81	89	-137	C2′-*endo*
48	m^5C	-109	178	76	141	-144	80	-145	C2′-*endo*
49	m^5C	58	158	40	85	-148	-70	-171	C3′-*endo*
50	U	-64	-178	46	81	-150	-74	-163	C3′-*endo*
51	G	-65	172	47	77	-160	-62	-166	C3′-*endo*
52	G	-84	-179	69	76	-150	-69	-172	C3′-*endo*
53	G	-64	174	64	77	-153	-71	-171	C3′-*endo*
54	s^2U	-60	180	54	82	-135	-65	-160	C3′-*endo*
55	Ψ	-73	168	59	78	-132	-77	-160	C3′-*endo*
56	Cm	-176	166	41	81	-138	-71	-162	C3′-*endo*
57	m^1I	-54	171	49	83	-143	-69	-152	C3′-*endo*
58	m^1A	-62	-139	62	149	-88	-176	-76	C2′-*endo*
59	G	69	-152	72	88	-153	-49	-177	C3′-*endo*
60	U	-74	-175	62	140	-96	-72	-124	C2′-*endo*
61	C	-80	-179	35	80	-154	-72	-168	C3′-*endo*
62	C	-72	-177	53	82	-146	-78	-167	C3′-*endo*
63	C	-53	175	44	80	-147	-71	-161	C3′-*endo*
64	A	-68	169	57	78	-155	-72	-166	C3′-*endo*
65	G	-65	174	56	79	-148	-71	-165	C3′-*endo*
66	C	-59	170	54	79	-149	-71	-160	C3′-*endo*
67	G	-64	167	56	79	-149	-76	-170	C3′-*endo*
68	G	-65	170	57	79	-141	-78	-170	C3′-*endo*
69	G	-64	160	65	76	-155	-85	-171	C3′-*endo*
70	C	-60	171	51	81	-147	-76	-161	C3′-*endo*
71	C	-63	172	50	80	-152	-68	-160	C3′-*endo*

72	C	-61	174	53	77	-147	-72	-165	C3′-*endo*
73	A	-65	167	60	78	-151	-74	-171	C3′-*endo*
74	C	-57	179	50	82	-140	-73	-167	C3′-*endo*
75	C	-52	168	47	82	-142	-63	-158	C3′-*endo*
76	A	-65	167	55	81	-	-	-158	C3′-*endo*

Supplementary Table 3b | Torsion angles and ribose puckering in *S. tokodaii* tRNA^{Val3} Molecule B.

Position	Nucleotide	α	β	γ	δ	ε	ζ	χ	puckering
1	G	-	-158	50	85	-153	-76	-168	C3′-*endo*
2	G	-67	175	55	80	-154	-68	-166	C3′-*endo*
3	G	-69	-179	57	79	-148	-79	-163	C3′-*endo*
4	C	-54	165	51	79	-144	-75	-164	C3′-*endo*
5	C	-56	164	62	80	-148	-72	-164	C3′-*endo*
6	ac^{4}C	-67	156	79	83	-148	-68	-170	C3′-*endo*
7	G	-65	-178	56	140	-90	-56	-131	C2′-*endo*
8	s^{4}U	-71	-168	60	83	-101	-145	-161	C3′-*endo*
9	C	-60	151	37	140	-96	-71	-97	C2′-*endo*
10	m^{2}G	169	170	58	83	-142	-76	-176	C3′-*endo*
11	U	-74	177	59	84	-150	-67	-158	C3′-*endo*
12	C	-66	175	47	80	-148	-79	-151	C3′-*endo*
13	Ψ	129	-138	-160	89	-143	-106	-175	C3′-*endo*
14	A	72	-174	-98	97	-133	-57	-165	C2′-*exo*
15	G$^+$	-61	166	62	87	-121	-75	-158	C3′-*endo*
16	C	-53	175	51	146	-94	-68	-110	C2′-*endo*
17	Cm	-54	152	60	79	-145	48	-154	C3′-*endo*
17a	U	169	152	43	149	-83	-117	-132	C2′-*endo*
18	G	56	163	41	153	-136	-99	-116	C2′-*endo*
19	G	-67	-164	36	142	-65	-113	-81	C2′-*endo*
20	U	-100	143	77	86	162	156	-110	C3′-*endo*
20a	U	-180	116	-61	142	-146	-78	-147	C2′-*endo*
21	A	115	146	47	82	-119	-150	-157	C3′-*endo*
22	G	-62	143	43	79	-148	-81	-167	C3′-*endo*
23	G	-52	169	50	80	-150	-78	-162	C3′-*endo*
24	A	-53	166	46	80	-146	-70	-157	C3′-*endo*
25	C	-67	164	58	76	-148	-63	-163	C3′-*endo*
26	m^{2,2}G	-69	-179	53	77	-142	-67	-171	C3′-*endo*
27	C	-63	-175	52	80	-135	-83	-175	C3′-*endo*
28	C	-62	167	52	77	-146	-81	-163	C3′-*endo*
29	G	-62	169	55	80	-154	-75	-166	C3′-*endo*
30	C	-67	171	57	82	-153	-70	-167	C3′-*endo*
31	C	-67	180	55	80	-148	-63	-168	C3′-*endo*
32	Cm	-68	174	54	84	-126	-85	-168	C3′-*endo*

33	U	-52	160	49	83	-115	-51	-154	C3′-*endo*
34	Um	-165	132	28	82	-112	-89	178	C3′-*endo*
35	A	-87	133	84	82	-105	-139	173	C3′-*endo*
36	C	105	-103	-170	83	-113	-91	173	C3′-*endo*
37	A	136	-132	164	83	-124	-71	170	C3′-*endo*
38	A	-77	170	55	81	-136	-74	-177	C3′-*endo*
39	G	-51	167	50	80	-142	-77	-174	C3′-*endo*
40	G	-54	172	47	82	-144	-84	-165	C3′-*endo*
41	C	-51	163	45	78	-138	-87	-161	C3′-*endo*
42	G	-41	158	41	73	-145	-79	-164	C3′-*endo*
43	G	-52	155	70	80	-153	-78	-176	C3′-*endo*
44	A	-62	-178	50	84	-139	-69	-162	C3′-*endo*
45	G	-60	164	54	85	-120	-76	-157	C3′-*endo*
46	G	-47	171	67	84	-145	-77	171	C3′-*endo*
47	U^{p}	-64	-172	59	140	-132	119	-137	C2′-*endo*
48	m^{5}C	-80	159	58	139	-151	77	-148	C2′-*endo*
49	m^{5}C	48	154	47	81	-144	-77	-172	C3′-*endo*
50	U	-56	173	44	79	-145	-73	-166	C3′-*endo*
51	G	-72	169	60	76	-153	-72	-166	C3′-*endo*
52	G	-73	169	65	76	-147	-73	-173	C3′-*endo*
53	G	-54	172	55	80	-152	-70	-171	C3′-*endo*
54	s^{2}U	-64	179	57	80	-128	-67	-162	C3′-*endo*
55	Ψ	-74	163	63	79	-134	-76	-159	C3′-*endo*
56	Cm	176	169	45	82	-134	-74	-167	C3′-*endo*
57	m^{1}I	-48	165	46	84	-145	-72	-155	C3′-*endo*
58	m^{1}A	-63	-142	63	150	-87	-175	-81	C2′-*endo*
59	G	71	-152	70	87	-149	-52	179	C3′-*endo*
60	U	-72	-172	63	140	-96	-74	-124	C2′-*endo*
61	C	-81	-175	34	84	-154	-72	-168	C3′-*endo*
62	C	-66	-176	48	82	-149	-72	-159	C3′-*endo*
63	C	-61	175	49	81	-159	-69	-157	C3′-*endo*
64	A	-71	179	53	80	-159	-67	-165	C3′-*endo*
65	G	-68	179	53	80	-147	-75	-164	C3′-*endo*
66	C	-53	165	49	79	-146	-76	-162	C3′-*endo*
67	G	-58	165	51	78	-153	-72	-169	C3′-*endo*
68	G	-65	177	55	76	-158	-68	-169	C3′-*endo*
69	G	-73	-179	61	79	-150	-77	-166	C3′-*endo*
70	C	-56	171	47	80	-153	-74	-161	C3′-*endo*

71	C	-65	170	59	78	-144	-77	-163	C3′-*endo*
72	C	-54	164	50	79	-151	-67	-164	C3′-*endo*
73	A	-70	175	59	80	-142	-65	-172	C3′-*endo*
74	C	-65	173	59	82	-141	-53	-172	C3′-*endo*
75	C	-88	176	69	87	-89	107	-155	C3′-*endo*
76	A	65	153	173	148	-	-	61	C2′-*endo*

Supplementary Table 3c | Torsion angles and ribose puckering in Tpt1p-treated tRNA Molecule A.

Position	Nucleotide	α	β	γ	δ	ε	ζ	χ	puckering
1	G	-	160	53	78	-139	-85	-178	C3'-*endo*
2	G	-51	164	53	78	-149	-75	-171	C3'-*endo*
3	G	-60	177	50	79	-141	-74	-166	C3'-*endo*
4	C	-64	166	67	82	-155	-83	-161	C3'-*endo*
5	C	124	-150	-157	84	-123	-97	-175	C3'-*endo*
6	ac^{4}C	-40	147	52	84	-155	-71	-163	C3'-*endo*
7	G	-58	176	58	135	-90	-57	-141	C2'-*endo*
8	s^{4}U	-74	-156	53	82	-109	-148	-159	C3'-*endo*
9	C	109	-152	-138	129	-100	-46	-122	C1'-*exo*
10	m^{2}G	-161	179	41	83	-168	-53	-169	C3'-*endo*
11	U	-86	-165	54	81	-156	-66	-162	C3'-*endo*
12	C	-65	173	54	78	-145	-76	-158	C3'-*endo*
13	Ψ	109	-139	-141	84	-153	-86	-175	C3'-*endo*
14	A	133	-175	-154	83	-131	-52	-170	C3'-*endo*
15	G$^+$	-63	170	56	86	-92	-85	-160	C3'-*endo*
16	C	-24	166	35	136	-94	-87	-85	C2'-*endo*
17	Cm	-60	147	61	78	-138	48	-156	C3'-*endo*
17a	U	165	154	39	147	-73	-132	-143	C2'-*endo*
18	G	43	169	52	158	-114	-134	-111	C2'-*endo*
19	G	8	167	-31	154	-81	-166	-73	C2'-*endo*
20	U	156	-162	-179	147	-90	-66	-6	C2'-*endo*
20a	U	-61	160	-60	140	-106	-108	-112	C2'-*endo*
21	A	64	-147	64	143	-159	-135	-117	C2'-*endo*
22	G	-150	117	133	85	-130	-79	174	C3'-*endo*
23	G	-62	177	49	85	-148	-75	-161	C3'-*endo*
24	A	-68	170	53	80	-138	-77	-164	C3'-*endo*
25	C	-60	160	54	78	-138	-80	-167	C3'-*endo*
26	m^{2,2}G	-38	159	45	78	-150	-65	-167	C3'-*endo*
27	C	-64	-170	42	82	-151	-65	-157	C3'-*endo*
28	C	-76	179	55	77	-138	-86	-160	C3'-*endo*
29	G	-38	156	45	76	-141	-79	-169	C3'-*endo*
30	C	-64	158	64	76	-148	-75	-166	C3'-*endo*
31	C	-59	173	53	83	-151	-55	-167	C3'-*endo*
32	Cm	-89	179	64	86	-132	-53	-162	C3'-*endo*

33	U	-90	-176	60	77	-105	-110	-144	C3'-*endo*
34	Um	97	-146	92	89	-115	-82	-114	C3'-*endo*
35	A	-82	164	51	75	168	-82	-157	C3'-*endo*
36	C	-153	-133	116	76	-125	-83	178	C3'-*endo*
37	A	-33	160	39	81	-136	-70	-169	C3'-*endo*
38	A	-61	164	54	81	-140	-60	180	C3'-*endo*
39	G	-87	-167	57	81	-144	-80	-169	C3'-*endo*
40	G	-54	168	53	80	-156	-71	-165	C3'-*endo*
41	C	-65	176	54	77	-143	-78	-168	C3'-*endo*
42	G	-55	167	56	76	-156	-70	-164	C3'-*endo*
43	G	-58	173	46	77	-148	-84	-162	C3'-*endo*
44	A	-53	154	65	83	-131	-69	-167	C3'-*endo*
45	G	-50	169	42	80	-138	-91	-157	C3'-*endo*
46	G	-80	-156	63	85	-135	-78	-113	C3'-*endo*
47	U	103	169	-41	149	-118	57	-107	C2'-*endo*
48	m^{5}C	177	-126	115	141	-144	69	-139	C2'-*endo*
49	m^{5}C	55	159	50	81	-142	-82	-174	C3'-*endo*
50	U	-50	171	38	81	-143	-84	-157	C3'-*endo*
51	G	-49	165	46	73	-148	-64	-169	C3'-*endo*
52	G	-69	178	52	80	-154	-69	-171	C3'-*endo*
53	G	-66	166	72	80	-153	-68	-175	C3'-*endo*
54	s^{2}U	-71	179	56	79	-129	-67	-165	C3'-endo
55	Ψ	-65	168	51	75	-135	-90	-151	C3'-*endo*
56	Cm	118	-179	105	75	-114	-81	-175	C3'-*endo*
57	m^{1}I	-41	164	35	80	-146	-77	-153	C3'-*endo*
58	m^{1}A	-54	-143	65	154	-95	178	-72	C2'-*endo*
59	G	76	-155	79	85	-143	-62	-180	C3'-*endo*
60	U	-57	172	53	141	-105	-72	-131	C2'-*endo*
61	C	-98	-169	45	83	-166	-62	-161	C3'-*endo*
62	C	-78	-162	48	81	-159	-69	-163	C3'-*endo*
63	C	-71	-173	53	80	-139	-70	-163	C3'-*endo*
64	A	-59	165	57	72	-155	-72	-166	C3'-*endo*
65	G	-67	169	64	76	-146	-78	-169	C3'-*endo*
66	C	-49	166	52	79	-140	-82	-161	C3'-*endo*
67	G	-55	152	56	75	-144	-70	-171	C3'-*endo*
68	G	-63	172	60	80	-152	-55	-165	C3'-*endo*
69	G	-134	139	131	81	-126	-107	175	C3'-*endo*
70	C	-31	148	35	80	-141	-73	-158	C3'-*endo*

71	C	-74	162	68	77	-149	-72	-169	C3'-*endo*
72	C	-70	-178	50	80	-142	-83	-164	C3'-*endo*
73	A	-34	154	48	78	-157	-62	-168	C3'-*endo*
74	C	-76	174	64	80	-144	-70	-161	C3'-*endo*
75	C	-72	-170	42	81	-148	-60	-169	C3'-*endo*
76	A	-73	167	71	83	-	-	-176	C3'-*endo*

Supplementary Table 3d | Torsion angles and ribose puckering in Tpt1p-treated tRNA Molecule B.

Position	Nucleotide	α	β	γ	δ	ε	ζ	χ	puckering
1	G	-	-150	47	79	-160	-69	-165	C3'-*endo*
2	G	-82	174	71	79	-139	-81	-175	C3'-*endo*
3	G	-48	164	52	81	-145	-85	-166	C3'-*endo*
4	C	-51	166	45	80	-148	-71	-166	C3'-*endo*
5	C	-71	161	76	79	-155	-65	-166	C3'-*endo*
6	ac^4C	-73	177	60	82	-154	-67	-169	C3'-*endo*
7	G	-62	177	61	137	-80	-61	-137	C2'-*endo*
8	s^4U	-64	-167	59	84	-118	-89	-165	C3'-*endo*
9	C	-166	110	175	138	-93	-33	-122	C2'-*endo*
10	m^2G	-169	147	66	83	-127	-75	173	C3'-*endo*
11	U	-69	164	61	80	-139	-92	-168	C3'-*endo*
12	C	-27	157	26	78	-163	-64	-158	C3'-*endo*
13	Ψ	126	-160	-156	81	-164	-64	-177	C3'-*endo*
14	A	155	166	-170	85	-122	-70	-172	C3'-*endo*
15	G^+	-43	152	50	82	-95	-100	-166	C3'-*endo*
16	C	-1	147	23	139	-95	-85	-85	C2'-*endo*
17	Cm	-49	149	60	79	-149	48	-162	C3'-*endo*
17a	U	154	155	53	149	-97	-104	-137	C2'-*endo*
18	G	79	151	26	152	-152	-92	-112	C2'-*endo*
19	G	-68	-155	35	145	-70	-105	-78	C2'-*endo*
20	U	-112	142	80	88	170	174	-124	C3'-*endo*
20a	U	174	124	-68	139	-135	-64	-154	C2'-*endo*
21	A	91	165	43	83	-157	-139	-154	C3'-*endo*
22	G	-29	143	37	81	-156	-70	-170	C3'-*endo*
23	G	-74	178	58	80	-154	-68	-173	C3'-*endo*
24	A	-70	179	55	84	-143	-73	-158	C3'-*endo*
25	C	-65	161	55	75	-135	-74	-165	C3'-*endo*
26	$m^{2,2}G$	-48	158	47	75	-134	-78	-170	C3'-*endo*
27	C	-49	169	44	79	-124	-93	-163	C3'-*endo*
28	C	-49	155	47	77	-148	-78	-167	C3'-*endo*
29	G	-67	177	52	83	-162	-69	-157	C3'-*endo*
30	C	-77	177	61	81	-143	-81	-164	C3'-*endo*
31	C	-49	169	46	83	-148	-71	-159	C3'-*endo*
32	Cm	-65	169	57	82	-122	-95	-165	C3'-*endo*
33	U	-46	153	47	82	-99	-48	-164	C3'-*endo*

34	Um	-137	114	27	80	-136	-85	-151	C3'-*endo*
35	A	-63	156	56	82	-130	-134	-148	C3'-*endo*
36	C	100	-99	-165	85	-107	-73	175	C3'-*endo*
37	A	97	-130	-154	84	-145	-50	-177	C3'-*endo*
38	A	-127	-163	70	78	-151	-56	-174	C3'-*endo*
39	G	-78	-175	71	79	-155	-60	-158	C3'-*endo*
40	G	-74	-177	52	78	-138	-94	-167	C3'-*endo*
41	C	-48	161	44	82	-153	-74	-165	C3'-*endo*
42	G	-61	171	56	77	-142	-75	-171	C3'-*endo*
43	G	-47	167	50	74	-169	-74	-163	C3'-*endo*
44	A	-73	179	63	84	-126	-75	-167	C3'-*endo*
45	G	-56	162	53	83	-141	-73	-155	C3'-*endo*
46	G	146	143	-143	110	-76	-133	-121	C2'-*exo*
47	U	104	-93	-109	135	-145	136	59	C2'-*endo*
48	m^5C	-22	141	16	139	-134	63	-141	C2'-*endo*
49	m^5C	83	142	27	80	-155	-68	-171	C3'-*endo*
50	U	-86	-172	62	82	-149	-70	-162	C3'-*endo*
51	G	-71	169	58	77	-158	-66	-167	C3'-*endo*
52	G	-77	172	63	75	-144	-70	-174	C3'-*endo*
53	G	-62	168	64	79	-145	-78	-175	C3'-*endo*
54	s^2U	-51	176	45	79	-132	-66	-148	C3'-*endo*
55	Ψ	-69	163	57	75	-121	-79	-158	C3'-*endo*
56	Cm	-160	159	36	83	-137	-74	-158	C3'-*endo*
57	m^1I	-55	167	42	84	-163	-62	-157	C3'-*endo*
58	m^1A	-78	-132	75	147	-90	-171	-83	C2'-*endo*
59	G	65	-156	84	88	-139	-65	179	C3'-*endo*
60	U	-51	170	49	141	-102	-69	-127	C2'-*endo*
61	C	-98	-174	45	80	-154	-73	-169	C3'-*endo*
62	C	-66	-178	47	81	-140	-81	-168	C3'-*endo*
63	C	-55	170	49	79	-154	-61	-164	C3'-*endo*
64	A	-76	169	67	75	-144	-80	-169	C3'-*endo*
65	G	-47	164	41	79	-142	-79	-168	C3'-*endo*
66	C	-52	159	52	79	-142	-73	-170	C3'-*endo*
67	G	-74	161	68	73	-126	-91	-176	C3'-*endo*
68	G	-55	151	65	79	-148	-75	-167	C3'-*endo*
69	G	-57	172	43	74	-159	-76	-164	C3'-*endo*
70	C	-74	168	65	75	-151	-69	-169	C3'-*endo*
71	C	-69	-178	56	79	-148	-70	-167	C3'-*endo*

72	C	-64	169	56	80	-131	-86	-165	C3'-*endo*
73	A	-43	151	54	78	-139	-70	-174	C3'-*endo*
74	C	-77	160	74	83	-119	-70	-159	C3'-*endo*
75	C	-63	157	56	83	-87	117	-161	C3'-*endo*
76	A	63	164	158	152	-	-	81	C2'-*endo*

Supplementary Table 3e | Torsion angles and ribose puckering in yeast tRNA^{Phe} (PDB: 1EHZ).

Position	Nucleotide	α	β	γ	δ	ε	ζ	χ	puckering
1	G	-	-128	68	83	-156	-69	-168	C3′-*endo*
2	C	-67	-178	54	83	-145	-77	-164	C3′-*endo*
3	G	-74	170	59	81	-148	-80	-162	C3′-*endo*
4	G	-64	162	61	82	-157	-69	-169	C3′-*endo*
5	A	-75	-176	53	85	-137	-82	-163	C3′-*endo*
6	U	-49	158	55	81	-151	-77	-160	C3′-*endo*
7	U	-59	-179	63	137	-106	-52	-133	C2′-*endo*
8	U	-84	-146	55	79	-143	-119	-162	C3′-*endo*
9	A	-70	-142	52	148	-106	-77	-71	C2′-*endo*
10	m^{2}G	178	147	60	89	-126	-89	170	C3′-*endo*
11	C	-56	168	48	87	-150	-70	-161	C3′-*endo*
12	U	-68	173	52	81	-159	-65	-158	C3′-*endo*
13	C	167	-170	179	83	-153	-97	-168	C3′-*endo*
14	A	83	-158	-115	92	-126	-57	-171	C2′-*exo*
15	G	-55	162	52	80	-136	-144	-165	C3′-*endo*
16	D	-6	91	77	97	-62	-131	-86	C3′-*endo*
17	D	28	108	174	95	178	76	-142	C2′-*exo*
18	G	45	-159	59	151	-95	-179	-100	C2′-*endo*
19	G	-71	-179	54	154	-92	-84	-80	C2′-*endo*
20	G	-81	-151	48	90	-122	-54	178	C3′-*endo*
21	A	-76	149	-177	78	-169	-76	-160	C3′-*endo*
22	G	159	153	179	82	-145	-80	-176	C2′-*exo*
23	A	-53	175	52	82	-155	-66	-158	C3′-*endo*
24	G	-69	178	47	84	-144	-73	-161	C3′-*endo*
25	C	-65	169	54	83	-145	-68	-160	C3′-*endo*
26	m^{2,2}G	-54	171	48	86	-136	-77	-163	C3′-*endo*
27	C	-53	167	44	83	-149	-73	-168	C3′-*endo*
28	C	-72	178	49	80	-152	-67	-161	C3′-*endo*
29	A	-67	174	56	81	-156	-78	-166	C3′-*endo*
30	G	-54	166	57	84	-145	-62	-172	C3′-*endo*
31	A	-70	178	52	84	-137	-76	-157	C3′-*endo*
32	Cm	-53	161	49	80	-146	-71	-150	C3′-*endo*
33	U	-68	-177	47	82	-148	-54	-148	C3′-*endo*
34	Gm	171	148	53	83	-133	-72	-171	C3′-*endo*

35	A	-48	164	40	81	-144	-60	-154	C3′-*endo*
36	A	-52	166	51	72	-160	-85	-158	C4′-*exo*
37	yW	-58	163	48	81	-148	-67	-169	C3′-*endo*
38	A	-62	-180	47	82	-137	-76	-169	C3′-*endo*
39	Ψ	-48	160	53	79	-140	-69	-166	C3′-*endo*
40	m^5C	-67	172	56	83	-154	-75	-163	C3′-*endo*
41	U	-68	-179	52	79	-137	-85	-169	C3′-*endo*
42	G	-48	159	56	80	-160	-70	-169	C3′-*endo*
43	G	-67	-178	56	82	-155	-76	-160	C3′-*endo*
44	A	-60	162	60	85	-143	-57	-159	C3′-*endo*
45	G	-72	-177	51	88	-135	-79	-149	C3′-*endo*
46	m^7G	-57	-147	48	142	-103	-138	-66	C2′-*endo*
47	U	62	-164	44	146	-94	-78	-112	C2′-*endo*
48	C	-73	-174	161	146	-144	76	-140	C2′-*endo*
49	m^5C	51	168	42	84	-145	-82	-174	C3′-*endo*
50	U	-52	177	42	80	-151	-68	-165	C3′-*endo*
51	G	-64	177	53	79	-150	-71	-157	C3′-*endo*
52	U	-65	174	49	80	-156	-69	-164	C3′-*endo*
53	G	-57	171	56	84	-159	-65	-169	C3′-*endo*
54	m^5U	-80	-173	58	78	-129	-71	-161	C3′-*endo*
55	Ψ	-50	169	44	77	-141	-70	-147	C3′-*endo*
56	C	166	172	53	83	-133	-71	-161	C3′-*endo*
57	G	-66	167	57	82	-145	-68	-159	C3′-*endo*
58	m^1A	-61	-146	72	157	-78	-169	-86	C2′-*endo*
59	U	73	-159	64	85	-149	-54	-166	C3′-*endo*
60	C	-72	179	66	148	-97	-66	-118	C2′-*endo*
61	C	-84	180	38	83	-152	-74	-167	C3′-*endo*
62	A	-60	180	47	81	-146	-74	-159	C3′-*endo*
63	C	-62	167	51	81	-152	-71	-153	C3′-*endo*
64	A	-67	180	44	76	-147	-77	-162	C3′-*endo*
65	G	-44	164	50	80	-152	-73	-173	C3′-*endo*
66	A	-58	179	52	82	-151	-74	-165	C3′-*endo*
67	A	-62	164	54	83	-152	-78	-163	C3′-*endo*
68	U	-60	175	47	82	-153	-65	-160	C3′-*endo*
69	U	-64	168	55	79	-155	-86	-161	C3′-*endo*
70	C	-62	165	53	79	-159	-65	-152	C3′-*endo*
71	G	-78	174	60	80	-150	-68	-163	C3′-*endo*
72	C	-73	176	62	83	-152	-68	-162	C3′-*endo*

73	A	-63	178	50	82	-148	-66	-167	C3′-*endo*
74	C	-67	-175	51	86	-145	-59	-153	C3′-*endo*
75	C	-52	176	42	86	-132	164	-152	C3′-*endo*
76	A	-71	130	165	161	-	-	138	C2′-*endo*

Supplementary Table 4 | List of S. tokodaii genes narrowed down by comparative genomics.

Gene names	Submitted protein names on UniPtotKB	COGs	Annotations of COGs*
STK_02355/sm2	Archaeal Sm protein	COG1958	Small nuclear ribonucleoprotein (snRNP) homolog
STK_04630	Polynucleotide 5'-hydroxyl-kinase	COG1341	Polynucleotide 5'-kinase, involved in rRNA processing
STK_04690	Sld5 domain-containing protein	COG1711	DNA replication initiation complex subunit, GINS family
STK_07800	Putative MFS transporter	COG0477	MFS family permease
STK_09530	Uncharacterized protien	COG2112	Predicted Ser/Thr protein kinase
STK_14200	Uncharacterized protien	COG4046	Uncharacterized protein
STK_14340/atpF	Putative membrane-associated ATPase F subunit	COG1436	Archaeal/vacuolar-type H+-ATPase subunit F/Vma7
STK_14405/rpls26e	30S ribosomal protein S26e	COG4830	Ribosomal protein S26
STK_20470	Uncharacterized protien	COG0500	SAM-dependent methyltransferase

* Annotations of COGs are reffered to the file "ftp://ftp.ncbi.nih.gov/pub/COG/COG2014/data/cognames2003-2014.tab".

Supplementary Table 5 | Partial sequences of *E. coli* tRNAs modified by NvArkI in the cell.

tRNA	Sequence (44 : 52)*
Ala1A, Ala1B	AGGUCUGCGG
Ala2	AGGUCAGCGG
Arg1, Arg2	CGGUCGGAGG
Asp	GGGUCGCGGG
Gly3	GGGUCGCGAG
His	UUGUCGUGGG
Ile1	AGGUCGGUGG
Ile2	UGGUCGCUGG
Lys	UGGUCGCAGG
fMet1	AGGUCGUCGG
Met	GGGUCACAGG
Pro1, Pro2, Pro3	GGGUCGGAGG
Thr2	AGGUCGUAGG
Thr3	AGGUCGGCAG
Val1	GGGUCGGCGG
Val2A	GGGUCGGUGG
Val2B	GGGUCGUUGG

*Primary sequence was extracted from positions 44 (V-loop) to 52 (T-stem).
RNA fragments generated by RNase T_1 are shown in red.

Supplementary Table 6 | List of probes and primers used in this study.

Experiment	Sequence (5′ - 3′)
**tRNA isolation**	
tRNA^{Val3}	5′ ECamino-AGGGCGGCGTCCTAACCAGGCTAGACGACGGGCCC-3′
tRNA^{Lys2}	5′ ECamino-GTTAAAAGCCCGCCGCTCTACCTGGCTGAGCTACGGGCCC-3′
tRNA^{Thr3}	5′ ECamino-TTACAAGGCCGGCGCTCTAACCAGGCTGAGCTACAGCGGC-3′
tRNA^{Gly3}	5′ ECamino-GGCCAGCGTCCTAATCCAGACTAGACGACGGCCGC-3′
tRNA^{Arg4}	5′ ECamino-TCCGAAGGCCGGCGCTCTATCCTGGCTGAGCTACGGGTCC-3′
tRNA^{Pro2}	5′ ECamino-GGCCTGCATCCTAGTCCAGGCTAGACGACGGTCCC-3′
tRNA^{Cys}	5′ ECamino-TGCGGCCCGCCGCCTTAACCTCTCGGCCACCCCGGC-3′
tRNA^{Gln2}	5′ ECamino-GGCCAAAGCCCTCGATCCTTGACCACTAGACGACCCGGCT-3′
tRNA^{Leu4}	5′ ECamino-CCTAAGCCCGACCCCTTTGACCTTGCTCGGGCACCCCCGC-3′
tRNA^{Ser3}	5′ ECamino-CTCTCAAGGCCAGCCCCTTAGTCCACTCGGGCACCCCGGC-3′
tRNA^{Ile2}	5′ ECamino-GAGCCGGGCGCTCTACCGGGCTAAGCTACGGGCCC-3′
tRNA^{Phe}	5′ ECamino-GTCTTCAGCCGGGCGCTCTCCCGGGCTGAGCTACG-3′
**Gene cloning**	
ScTpt1_Fw	5′-GACTGACTCATATGCGCCAGGTACTACAAAA-3′
ScTpt1_Rv	5′-GACTCTCGAGTATCTTTTCGAGCGGTATGTTTC-3′
TkArkI_Fw	5′-AAACTTGTACTTCCAAGGAATGACCTTCGAGCATATC-3′
TkArkI_Rv	5′-ATTCGGATCCCATATGGGATCACAGACCGCTCAGTAAAG-3′
MfArkI_Fw	5′-AAACTTGTACTTCCAAGGAATGGCCATTAAAAAAGAAATTC-3′
MfArkI_Rv	5′-ATTCGGATCCCATATGGGATCACAGTTTTTTATAGGTTTTGGC-3′

NvArkI_Fw	5′-GTGATCTGGGATCCATGTCTCAACAGAGAGCC-3′
NvArkI_Rv	5′-GTGATCTGGCGGCCGCTCAGGCAACAACAACAAG-3′
AaArkI_Fw	5′-AAACTTGTACTTCCAAGGAATGAAATTTAGCGAGTTCATTA-3′
AaArkI_Rv	5′-ATTCGGATCCCATATGGGATCAGCTCAGCACTTTGCG-3′
NpArkI_Fw	5′-CCGCGAACAGATTGGAGGTATGATCATCTTCGAGAAAAAGGG-3′
NpArkI_Rv	5′-CTTGGAAGTACAAGTTTTCTCAGCTTTCATCAATCAGGCGAATA-3′
LeArkI_Fw	5′-CCGCGAACAGATTGGAGGTATGGCAGGTAGCCAGTTTC-3′
LeArkI_Rv	5′-CTTGGAAGTACAAGTTTTCTCACAGATTCAGGCTGCTCAG-3′
TkKptA_Fw	5′-AAACTTGTACTTCCAAGGAATGAAGCCAGAGCGGAAG-3′
TkKptA_Rv	5′-ATTCGGATCCCATATGGGACTAAACCGCCAGGGTTATACA-3′
pSUMO_Fw	5′-TCCCATATGGGATCCG-3′
pSUMO_Rv	5′-TCCTTGGAAGTACAAGTTT-3′
pSUMO_Fw2	5′-GAAAACTTGTACTTCCAAGGAT-3′
pSUMO_Rv2	5′-ACCTCCAATCTGTTCGCG-3′
**Gene disruption**	
TkArkI_Upstream_pUD3_Fw	5′-GCTTGCATGCCTGCAGTCCTTCCATAGGTAGATG-3′
TKArkI_Upstream_Rv	5′-ACTATTTAAGCTTATCGCGTCAAGAGAACCGGAAGCCTC-3′
TkArkI_Downstream_Fw	5′-ACGCGATAAGCTTAAATAGT-3′
TkArkI_Downstream_pUD3_Rv	5′-CCTCTAGAGTCGACCAGTCCAAACCCTCGATATAA-3′
TkKptA_Upstream_pUD3_Fw	5′-CCAAGCTTGCATGCCTGCAACCATCCGCAAAGGGGAG-3′
TkKptA_Upstream_Rv	5′-GAAACTCGCATGGGCTGACGCTAAAAACCTCACGGGA-3′
TkKptA_Downstream_Fw	5′-GTCAGCCCATGCGAGTTTC-3′
TkKptA_Downstream_pUD3_Rv	5′-GGATCCTCTAGAGTCGACCCTTTGCTATCAAAAGCCGC-3′

pUD3_Fw	5′-GGTCGACTCTAGAGGATCCC-3′
pUD3_Rv	5′-TGCAGGCATGCAAGCTTG-3′
***in vitro* T7 transcription**	
Tk tRNA^{Val3}_Fw	5′-CAGTAATACGACTCACTATAGGGCCCGTGGTCTAGATGGTT-3′
Tk tRNA^{Val3}_Body	5′-CCGTGGTCTAGATGGTTATGACGCCACCCTTACAAGGTGGAGGTCCGG-3′
Tk tRNA^{Val3}_Rv	5′-TGGTGGGCCCGCGGGGATTCGAACCCCGGACCTCCACCTTG-3′
Tk tRNA^{Val3}_G5:C68_Fw	5′-CAGTAATACGACTCACTATAGGGCGCGTGGTCTAGATG-3′
Tk tRNA^{Val3}_G5:C68_Body	5′-<br>AGGGCGCGTGGTCTAGATGGTTATGACGCCACCCTTACAAGGTGGAGGTCCGGGGTT-3′
Tk tRNA^{Val3}_G5:C68_Rv	5′-TGGTGGGCGCGCGGGGATTCGAACCCCGGACCTCCACCTT-3′
**Mutation study**	
TkArkI_K32A_Fw	5′-GCTGGCAGCGGGTACCACCTCTTTAGTG -3′
TkArkI_K32A_Rv	5′-GTACCCGCTGCCAGCGGTTCCACACCAC -3′
TkArkI_G33A_Fw	5′-GCAAAGGCGACCACCTCTTTAGTGTTTAC-3′
TkArkI_G33A_Rv	5′-GGTGGTCGCCTTTGCCAGCGGTTCCAC-3′
TkArkI_K51A_Fw	5′-GGTGATTGCGCTGCAGCGCCCGGACAGC-3′
TkArkI_K51A_Rv	5′-GCTGCAGCGCAATCACCACTTTGCGACC-3′
TkArkI_E65A_Fw	5′-GAAAAAGCAGCTGAGCTGACCAAAATC-3′
TkArkI_E65A_Rv	5′-CTCAGCTGCTTTTTCAAAATTGCTACG -3′
TkArkI_R95A_Fw	5′-CTGATTGCGGAATTTGCCGAGGGCGAAC-3′
TkArkI_R95A_Rv	5′-CAAATTCCGCAATCAGATACGGTAAACC-3′
TkArkI_H130A_Fw	5′-ATTGATGCTGGCCAGATCCAAGGTGGC-3′
TKArkI_H130A_Rv	5′-CTGGCCAGCATCAATGCCCAGACGATC-3′
TkArkI_Q132A_Fw	5′-CATGGCGCGATCCAAGGTGGCAAACAC-3′

TkArkI_Q132A_Rv	5′-TTGGATCGCGCCATGATCAATGCCCAG-3′
TkArkI_K137A_Fw	5′-GGTGGCGCGCACATTATCATCGGCGAG-3′
TkArkI_K137A_Rv	5′-AATGTGCGCGCCACCTTGGATCTGGCC-3′
TkArkI_D149A_Fw	5′-CTGATCGCGTTTGAGAAGGCCGGCTTC-3′
TkArkI_D149A_Rv	5′-CTCAAACGCGATCAGATACACATCCTC-3′
TkArkI_N160A_Fw	5′-CCGAACGCGCTGACCAGCGCCATGGCC-3′
TkArkI_N160A_Rv	5′-GGTCAGCGCGTTCGGTTTGCGGAAGCC-3′
TkArkI_T162A_Fw	5′-AATCTGGCGAGCGCCATGGCCATGATCT-3′
TkArkI_T162A_Rv	5′-GGCGCTCGCCAGATTGTTCGGTTTGCG-3′
TkArkI_Y200A_Fw	5′-CGCCACGCGAAACGCACCGGTAGTCTG-3′
TkArkI_Y200A_Rv	5′-GCGTTTCGCGTGGCGTAAAGCATCTTTC-3′
TkArkI_K201A_Fw	5′-CACTACGCGCGCACCGGTAGTCTGAGT -3′
TkArkI_K201A_Rv	5′-GGTGCGCGCGTAGTGGCGTAAAGCATC-3′
TkArkI_R202A_Fw	5′-ACTACAAAGCGACCGGTAGTCTGAGTCGT-3′
TkArkI_R202A_Rv	5′-ACCGGTCGCTTTGTAGTGGCGTAAAGC-3′

Supplementary Table 7 | Strains and plasmids used in this study.

strain/plasmid	genotype/characteristics	source/reference
***Escherichia coli* strains**		
DH5a	F- φ80*lac*ZΔM15 Δ(*lac*ZYA-*arg*F)U169[*] *rec*A1 *end*A1 *hsd*R17(rK-, mK+) *pho*A *sup*E44 λ– *thi*-1 *gyr*A96 *rel*A1	
BL21(DE3)	F- *ompT hsdS*B(rB- mB-) *gal dcm* (DE3)	Merck
Rosetta2(DE3)	F- *ompT hsdS*B(rB- mB-) *gal dcm* (DE3) pRARE2 (Cam^r)[*]	Novagen
Δ*trmB*/Δ*tapT*	Δ*tapT::*Kan^r[*] Δ*trmB rrnB* Δ*lacZ*4787 *hsdR514* Δ*(araBAD)567* Δ*(rhaBAD)568 rph-1*	Laboratory stock
Δ*trmB*/Δ*tapT*/p*nvarkI*	Δ*tapT::*Kan^r Δ*trmB rrnB* Δ*lacZ*4787 *hsdR514* Δ*(araBAD)567* Δ*(rhaBAD)568 rph-1* pMW-J23106-*nvarkI*	this work
Δ*trmB*/Δ*tapT*/p*nvarkI*/p*tkkptA*	Δ*tapT::*Kan^r Δ*trmB rrnB* Δ*lacZ*4787 *hsdR514* Δ*(araBAD)567* Δ*(rhaBAD)568 rph-1* pMW-J23106-*nvarkI* pQE-80LC-*tkkptA*	this work
Δ*trmB*/Δ*tapT*/p*nvarkI*/p*eckptA*	Δ*tapT::*Kan^r Δ*trmB rrnB* Δ*lacZ*4787 *hsdR514* Δ*(araBAD)567* Δ*(rhaBAD)568 rph-1* pMW-J23106-*nvarkI* pQE-80LC-*eckptA*	this work
Δ*trmB*/Δ*tapT*/p*nvarkI*/p*sctpt1*	Δ*tapT::*Kan^r Δ*trmB rrnB* Δ*lacZ*4787 *hsdR514* Δ*(araBAD)567* Δ*(rhaBAD)568 rph-1* pMW-J23106-*nvarkI* pQE-80LC-*sctpt1*	this work
***Thermococcus kodakarensis* strains**		
KU216	Δ*pyrF* (uracil auxotroph) in KOD1 strain (wild type)	Sato *et al.* [50]
KD2239	Δ*pyrF* Δ*tk2239*	Orita *et al.* [19]
Δ*arkI*	Δ*pyrF* Δ*arkI*	this work
FFH05	Δ*pyrF* Δ*tk2239* Δ*queE*(*queE*::Tn)	Orita *et al.* [19]
Δ*arkI*/Δ*queE*	Δ*pyrF* Δ*tk2239* Δ*queE*(*queE*::Tn) Δ*arkI*	this work

plasmid

pET-21b	Expression vector; Kan^r	Merck
pET-21b-*sctpt1*	Expression vector; *tpt1 gene from S. cerevisiae* was inserted to pET-21b	this work
pE-SUMO-TEV	Expression vector; pE-SUMO Vector (LifeSensors), TEV-protease site is inserted between N-terminal SUMO-tag and MCS. Kan^r	Kato *et al.* [87)]
pE-SUMO-TEV-*tkarkI*	Expression vector; *arkI* gene from *T. kodakarensis* was inserted to pE-SUMO-TEV	this work
pE-SUMO-TEV-*mfarkI*	Expression vector; *arkI* gene from *M. fervens* was inserted to pE-SUMO-TEV	this work
pE-SUMO-TEV-*aaarkI*	Expression vector; *arkI* gene from *A. aeolicus* was inserted to pE-SUMO-TEV	this work
pE-SUMO-TEV-*nvarkI*	Expression vector; *arkI* gene from *N. viennensis* was inserted to pE-SUMO-TEV	this work
pE-SUMO-*nparkI*	Expression vector; *arkI* gene from *N. profundicola* was inserted to pE-SUMO-TEV	this work
pE-SUMO-*learkI*	Expression vector; *arkI* gene from *Leptolyngbya sp.* PCC7376 was inserted to pE-SUMO-TEV	this work
pE-SUMO-TEV-*tkkptA*	Expression vector; *kptA* gene from *T. kodakarensis* was inserted to pE-SUMO-TEV	this work
pMW118	Expression vector; Amp^r *	Invitrogen
pMW-J23106-*nvarkI*	Expression vector; synthetic promoter J23106 and *arkI* gene from *N. viennensis* with C-terminus His_6-tag and 3x FLAG was inserted to pMW118	this work
pQE-80L	Expression vector; Amp^r	QIAGEN
pACYC-RIL	Used for cloning of Cam^r	Stratagene
pQE-80LC-*tkkptA*	Expression vector; *kptA* gene from *T. kodakarensis* was inserted to pQE-80L and Amp^r was replaced to Cam^r	this work
pQE-80LC-*eckptA*	Expression vector; *kptA* gene from *E. coli* was inserted to pQE-80L and Amp^r was replaced to Cam^r	this work
pQE-80LC-*sctpt1*	Expression vector; *kptA* gene from *S. cerevisiae* was inserted to pQE-80L and Amp^r was replaced to Cam^r	this work
pUD3	*pyrF* marker cassette for *T. kodakarensis* was inserted in pUC118	Kobori *et al.* [66)]

pUD3-ArkI	Knockout vector; upstream region and downstream region of *arkI* gene from *T. kodakarensis* genome was inserted in pUD3	this work

* Resistance gene abbreviations as follows: Kan^r, Kanamycin resistance; Amp^r, Ampicillin resistance; Cam^r, Chloramphenicol resistance; Δ, deletion.

Supplementary Video

Supplementary Video 1 | A comparison of the base-triple in *S. tokodaii* tRNA^{Val3} Molecule A and Molecule B.

An animated image of the base-triple Ψ13–G22–G46/C9 in the core region of Mol. A and Mol. B generated by morphing with RigiMOL. Dashed lines indicate predicted interactions. U^{p}47 is shown in red.

Supplementary Reference

69 Culver, G. M. *et al.* An NAD derivative produced during transfer RNA splicing: ADP-ribose 1"-2" cyclic phosphate. *Science* 261, 206-208 (1993).

70 Taniguchi, T. *et al.* Acetate-dependent tRNA acetylation required for decoding fidelity in protein synthesis. *Nat Chem Biol* 14, 1010-1020, doi:10.1038/s41589-018-0119-z (2018).

71 Stern, L. & Schulman, L. H. The role of the minor base N^4-acetylcytidine in the function of the Escherichia coli noninitiator methionine transfer RNA. *J Biol Chem* 253, 6132-6139 (1978).

72 Yamaizumi, Z. *et al.* Archaebacterial tRNA contains 1-methylinosine at residue 57 in T psi C-loop. *Nucleic Acids Symp Ser*, 209-213 (1982).

73 Dixit, S., Henderson, J. C. & Alfonzo, J. D. Multi-Substrate Specificity and the Evolutionary Basis for Interdependence in tRNA Editing and Methylation Enzymes. *Front Genet* 10, 104, doi:10.3389/fgene.2019.00104 (2019).

74 Hori, H. Regulatory Factors for tRNA Modifications in Extreme- Thermophilic Bacterium Thermus thermophilus. *Front Genet* 10, 204, doi:10.3389/fgene.2019.00204 (2019).

75 Hatefi, Y. The mitochondrial electron transport and oxidative phosphorylation system. *Annu Rev Biochem* 54, 1015-1069, doi:10.1146/annurev.bi.54.070185.005055 (1985).

76 Cohen, P. The origins of protein phosphorylation. *Nat Cell Biol* 4, E127-130, doi:10.1038/ncb0502-e127 (2002).

77 Amitsur, M., Levitz, R. & Kaufmann, G. Bacteriophage T4 anticodon nuclease, polynucleotide kinase and RNA ligase reprocess the host lysine tRNA. *EMBO J* 6, 2499-2503 (1987).

78 Zillmann, M., Gorovsky, M. A. & Phizicky, E. M. Conserved mechanism of tRNA splicing in eukaryotes. *Mol Cell Biol* 11, 5410-5416, doi:10.1128/mcb.11.11.5410-5416.1991 (1991).

79 Weitzer, S. & Martinez, J. The human RNA kinase hClp1 is active on 3' transfer RNA exons and short interfering RNAs. *Nature* 447, 222-226, doi:10.1038/nature05777 (2007).

80 Salzman, D. W. *et al.* miR-34 activity is modulated through 5'-end phosphorylation in response to DNA damage. *Nat Commun* 7, 10954, doi:10.1038/ncomms10954 (2016).

81 Minvielle-Sebastia, L., Preker, P. J., Wiederkehr, T., Strahm, Y. & Keller, W. The

major yeast poly(A)-binding protein is associated with cleavage factor IA and functions in premessenger RNA 3'-end formation. *Proc Natl Acad Sci U S A* 94, 7897-7902, doi:10.1073/pnas.94.15.7897 (1997).

82 Gasse, L., Flemming, D. & Hurt, E. Coordinated Ribosomal ITS2 RNA Processing by the Las1 Complex Integrating Endonuclease, Polynucleotide Kinase, and Exonuclease Activities. *Mol Cell* 60, 808-815, doi:10.1016/j.molcel.2015.10.021 (2015).

83 Heindl, K. & Martinez, J. Nol9 is a novel polynucleotide 5'-kinase involved in ribosomal RNA processing. *EMBO J* 29, 4161-4171, doi:10.1038/emboj.2010.275 (2010).

84 Kurata, T. *et al.* RelA-SpoT Homolog toxins pyrophosphorylate the CCA end of tRNA to inhibit protein synthesis. *Mol Cell* 81, 3160-3170 e3169, doi:10.1016/j.molcel.2021.06.005 (2021).

85 Osawa, T. *et al.* Structural basis of tRNA agmatinylation essential for AUA codon decoding. *Nat Struct Mol Biol* 18, 1275-1280, doi:10.1038/nsmb.2144 (2011).

86 Terasaka, N., Kimura, S., Osawa, T., Numata, T. & Suzuki, T. Biogenesis of 2-agmatinylcytidine catalyzed by the dual protein and RNA kinase TiaS. *Nat Struct Mol Biol* 18, 1268-1274, doi:10.1038/nsmb.2121 (2011).

87 Kato, K. *et al.* Structural and functional analyses of DNA-sensing and immune activation by human cGAS. *PLoS One* 8, e76983, doi:10.1371/journal.pone.0076983. (2013).
