# Supplementary material for: Reversible RNA phosphorylation stabilizes tRNA for cellular thermotolerance
Source: Nature. 2022 Apr 27;605(7909):372–9. doi: 10.1038/s41586-022-04677-2 (PMC9095486; doi:10.1038/s41586-022-04677-2)
Supplement: Supplementary file 2 — Reporting Summary [file 41586_2022_4677_MOESM2_ESM.pdf]

Corresponding author(s): Tsutomu Suzuki
Last updated by author(s): Feb 8, 2022

Reporting Summary

Nature Portfolio wishes to improve the reproducibility of the work that we publish. This form provides structure for consistency and transparency in reporting. For further information on Nature Portfolio policies, see our Editorial Policies and the Editorial Policy Checklist.

Statistics

For all statistical analyses, confirm that the following items are present in the figure legend, table legend, main text, or Methods section.

n/a	Confirmed	
☐	☒	The exact sample size (n) for each experimental group/condition, given as a discrete number and unit of measurement
☐	☒	A statement on whether measurements were taken from distinct samples or whether the same sample was measured repeatedly
☐	☒	The statistical test(s) used AND whether they are one- or two-sided<br>*Only common tests should be described solely by name; describe more complex techniques in the Methods section.*
☒	☐	A description of all covariates tested
☒	☐	A description of any assumptions or corrections, such as tests of normality and adjustment for multiple comparisons
☐	☒	A full description of the statistical parameters including central tendency (e.g. means) or other basic estimates (e.g. regression coefficient) AND variation (e.g. standard deviation) or associated estimates of uncertainty (e.g. confidence intervals)
☐	☒	For null hypothesis testing, the test statistic (e.g. F, t, r) with confidence intervals, effect sizes, degrees of freedom and P value noted<br>*Give P values as exact values whenever suitable.*
☒	☐	For Bayesian analysis, information on the choice of priors and Markov chain Monte Carlo settings
☒	☐	For hierarchical and complex designs, identification of the appropriate level for tests and full reporting of outcomes
☒	☐	Estimates of effect sizes (e.g. Cohen's d, Pearson's r), indicating how they were calculated

Our web collection on statistics for biologists contains articles on many of the points above.

Software and code

Policy information about availability of computer code

Data collection	Gel images were obtained by FLA-7000 and FLA-9000.<br>Melting curves were obtained by V-630.<br>MS data were obtained by LTQ Orbitrap XL, Q Exactive Hybrid Quadrupole-Orbitrap Mass Spectrometer, LCQ-Advantage Ion-trap Mass Spectrometer.<br>Radioactivities were measured by Tri-Carb 2910TR1<br>Cell densities were monitored by S1200 diode array spectrophotometer.<br>X-ray diffraction data were obtained by Dectris Eiger X16MS detector and UGUA control system with BL17A beamline at the Photon Factory.

Data analysis

Canvas X (version 20), ACD/ChemSketch (Freeware, 2018.2.1) , ChemDraw (20.1.1), Excel (2016, 2019) and R (4.1.2) were used to draw figures.
Xcalibur (4.1) was used for mass spec analysis.
Spectra Manager (v2) was used for Tm measurement.
Multi Gauge (V3.0) was used for graphical analysis.
Prism 7 was used for kinetic analysis.
XDS/XSCALE (VERSION Feb 5, 2021) , SHELX (2016/1), Phaser (2.8.3), RESOLVE (2.15), APBS (1.5), Coot (0.8.9.1),and Phenix (Version 1.18.2-3874) were used for
structural analyses.
Coot (0.8.9.1), Pymol (2.4.0), Cuemol (2.2.3.443) were used for draw structure data.
RECOG (1.1.32) was used for comparative genome.
x3dna-dssr (v1.9.10) was used for analysis of torsion angles.
PhyloT (v2) and iTOL (6.5) were used for analysis of phylogenetic distribution.
DALI (v.5) and MAFFT (Version 7) were used for sequence alignment.

For manuscripts utilizing custom algorithms or software that are central to the research but not yet described in published literature, software must be made available to editors and reviewers. We strongly encourage code deposition in a community repository (e.g. GitHub). See the Nature Portfolio guidelines for submitting code & software for further information.

Data

Policy information about availability of data

All manuscripts must include a data availability statement. This statement should provide the following information, where applicable:

- Accession codes, unique identifiers, or web links for publicly available datasets
- A description of any restrictions on data availability
- For clinical datasets or third party data, please ensure that the statement adheres to our policy

Public databases: Microbial Genome Database(MBGD), NCBI database, COG database, BacDive, Genome Online Database, IMG database, Mongo Oligo Mass Calculator v2.08, Genomic tRNA database, Modomics, Protein Data Bank (PDB) (1EHZ, 1IVS, and 1ATP). Coordinates and structure factors have been deposited in PDB under accession code 7VNV, 7VNW, and 7VNX.

Field-specific reporting

Please select the one below that is the best fit for your research. If you are not sure, read the appropriate sections before making your selection.

☒ Life sciences ☐ Behavioural & social sciences ☐ Ecological, evolutionary & environmental sciences

For a reference copy of the document with all sections, see nature.com/documents/nr-reporting-summary-flat.pdf

Life sciences study design

All studies must disclose on these points even when the disclosure is negative.

Sample size	3 data points were used in student t-test.
Data exclusions	No data were excluded.
Replication	All attempts to replicate experiments succeeded.<br>Tm measurement, RNase probing, growth comparison, in vitro biochemical studies, were technically or biologically triplicated.
Randomization	Randomization was irrelevant for this basic study because it did not involve clinical trials or population studies.
Blinding	No blinding was required because the results of measurement or analysis was not affected by knowledge of sample identities.

Reporting for specific materials, systems and methods

We require information from authors about some types of materials, experimental systems and methods used in many studies. Here, indicate whether each material, system or method listed is relevant to your study. If you are not sure if a list item applies to your research, read the appropriate section before selecting a response.

Materials & experimental systems

n/a	Involved in the study
☒	☐ Antibodies
☒	☐ Eukaryotic cell lines
☒	☐ Palaeontology and archaeology
☒	☐ Animals and other organisms
☒	☐ Human research participants
☒	☐ Clinical data
☒	☐ Dual use research of concern

Methods

n/a	Involved in the study
☒	☐ ChIP-seq
☒	☐ Flow cytometry
☒	☐ MRI-based neuroimaging
